# Anti-OmpC antibodies in Crohn’s disease and ulcerative colitis: evidence from a systematic review and meta-analysis

**DOI:** 10.1093/crocol/otag056

**Published:** 2026-06-12

**Authors:** Ramy Sekla, Aleena Sammar, Amil Shah, Funmilayo Oludiran, Muhammad Sohaib, Mena Tawfik

**Affiliations:** Department of Medicine, UCHealth Parkview Medical Center, Pueblo, CO, United States; Department of Medicine, UCHealth Parkview Medical Center, Pueblo, CO, United States; Department of Medicine, UCHealth Parkview Medical Center, Pueblo, CO, United States; Department of Medicine, UCHealth Parkview Medical Center, Pueblo, CO, United States; Department of Medicine, UCHealth Parkview Medical Center, Pueblo, CO, United States; Department of Medicine, UCHealth Parkview Medical Center, Pueblo, CO, United States

**Keywords:** Crohn’s disease, ulcerative colitis, outer membrane porin C, biomarkers, serology, diagnostic biomarker, prognostic marker

## Abstract

**Objective:**

Anti-OmpC antibodies target the *Escherichia coli* outer membrane porin C. Their diagnostic and prognostic roles in inflammatory bowel disease (IBD) remain uncertain. We systematically assessed their prevalence, diagnostic accuracy, and clinical associations in Crohn’s disease (CD) and ulcerative colitis (UC).

**Methods:**

We performed a systematic review and meta-analysis (PROSPERO CRD420251066959) of 31 studies including ∼11 000 participants with CD, UC, and controls. Random-effects models estimated pooled prevalence, odds ratios (OR), sensitivity, specificity, diagnostic odds ratios (DOR), and summary receiver operating characteristic curves. Subgroup and sensitivity analyses were performed by assay cutoff, and publication bias was evaluated with funnel plots, Egger’s regression, and trim-and-fill.

**Results:**

For CD, 11 studies compared patients with controls; 8 using standardized ELISA cutoffs showed a strong association (OR = 6.99, 95% CI 3.99-12.23; I^2^ = 58%). For UC, 11 studies yielded OR = 4.75 (95% CI 2.82-7.99; I^2^ = 37%). Pooled prevalence was 36.9% in CD and 26.8% in UC. Diagnostic accuracy showed modest sensitivity (CD 0.39; UC 0.30) but high specificity (CD 0.89; UC 0.88), with area under the curve values of 0.76 and 0.77. In CD, OmpC positivity was associated with complicated phenotype (OR = 2.77) and surgery (OR = 2.41), but not with location, activity, or extra-intestinal manifestations.

**Conclusions:**

Anti-OmpC antibodies are strongly associated with CD and show a supportive but less consistent association in UC, with high specificity but limited sensitivity. In CD, OmpC positivity is associated with complicated disease behavior and increased odds of surgery, although these findings should be interpreted cautiously given the observational nature of the included studies. Incorporation into multi-marker serological panels may enhance diagnostic confidence and risk stratification.

## Graphical Abstract

**Graphical Abstract:**
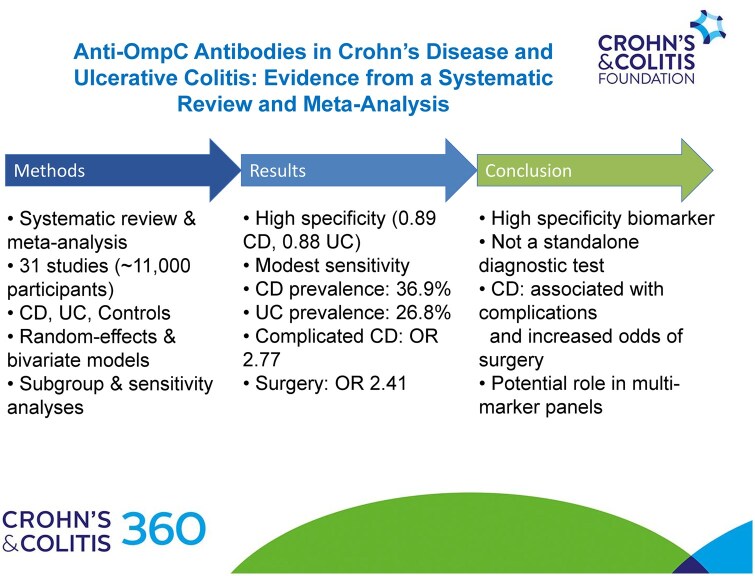

Key Messages **What is already known on this topic** 

Anti-OmpC antibodies have been studied in inflammatory bowel disease, but findings on prevalence, diagnostic accuracy, and clinical associations have been inconsistent.

**What this study adds** 

This systematic review and meta-analysis of 31 studies (∼11 000 participants) demonstrates that anti-OmpC antibodies have high specificity but modest sensitivity in both Crohn’s disease (CD) and ulcerative colitis, and that OmpC positivity in CD is associated with complicated phenotypes and increased likelihood of surgery.

**How this study might affect research, practice, or policy** 

Anti-OmpC testing may be associated with more aggressive CD phenotypes, and incorporation into multi-marker panels with anti-*Saccharomyces cerevisiae* antibodies (ASCA) and perinuclear anti-neutrophil cytoplasmic antibodies may enhance diagnostic confidence and support personalized risk stratification in IBD.

## Introduction

### Rationale

Serological antibodies are well-established biomarkers in inflammatory bowel disease (IBD). Anti-*Saccharomyces cerevisiae* antibodies (ASCA) and perinuclear anti-neutrophil cytoplasmic antibodies (pANCA) have been incorporated into diagnostic algorithms,^1^^,^^2^ yet other microbial-directed antibodies, including anti-OmpC, remain less well characterized.

Individual studies have reported associations between anti-OmpC antibodies and IBD, particularly Crohn’s disease (CD),^3–11^ but findings have been inconsistent. Prevalence varies across populations and assay cutoffs, and the clinical significance in ulcerative colitis (UC) is even less clear.^12–15^ Moreover, while some studies suggest associations with complicated CD phenotypes or surgical outcomes, others report null results.^3^^,^^16^

Mechanistically, anti-OmpC differs from ASCA and pANCA in its immunologic target. ASCA reflects an abnormal response to yeast cell wall components, and pANCA represents autoimmunity against neutrophil antigens. In contrast, anti-OmpC arises from immune recognition of *Escherichia coli* outer membrane porin C, suggesting a more direct link to microbial dysbiosis and host–pathogen interactions. This distinction may explain why OmpC has consistently shown stronger prognostic rather than diagnostic associations, particularly in CD, where bacterial-driven immune responses play a central pathogenic role.

Given these discrepancies, the clinical utility of anti-OmpC antibodies in IBD remains uncertain. Importantly, no prior meta-analysis has comprehensively synthesized prevalence, diagnostic accuracy, and clinical associations in both CD and UC.

### Objective

To systematically review and meta-analyze studies of anti-OmpC antibodies in IBD, estimating pooled prevalence, diagnostic accuracy, and associations with clinical outcomes including complicated CD, surgery, disease location, activity, and extra-intestinal manifestations.

## Methods

### Protocol and reporting

This review was conducted in accordance with PRISMA 2020 guidelines^17^ and registered with PROSPERO (CRD420251066959).^18^

### Literature search

A comprehensive search was conducted in PubMed, VHL Regional Portal, Web of Science, Cochrane Library, and Google Scholar from database inception through May 5, 2025. Search strategies combined disease-related terms (“inflammatory bowel disease,” “Crohn’s disease,” “ulcerative colitis”) with antibody-related terms (“anti-OmpC,” “outer membrane porin C,” “*Escherichia coli* outer membrane porin C antibodies”).

The searches yielded a total of 2037 records: 82 from PubMed, 102 from VHL, 87 from Web of Science, 4 from Cochrane, and 1762 from Google Scholar. Before screening, 185 duplicates and 308 records without available abstracts were removed, leaving 1544 records for title and abstract screening. Of these, 117 full-text articles were retrieved and assessed for eligibility. After excluding 86 studies (conference abstracts, no extractable OmpC data, unavailable full text, duplicates, non-original studies, or non-IBD populations), 31 studies met inclusion criteria and were included in the final synthesis.

**Figure 1:**
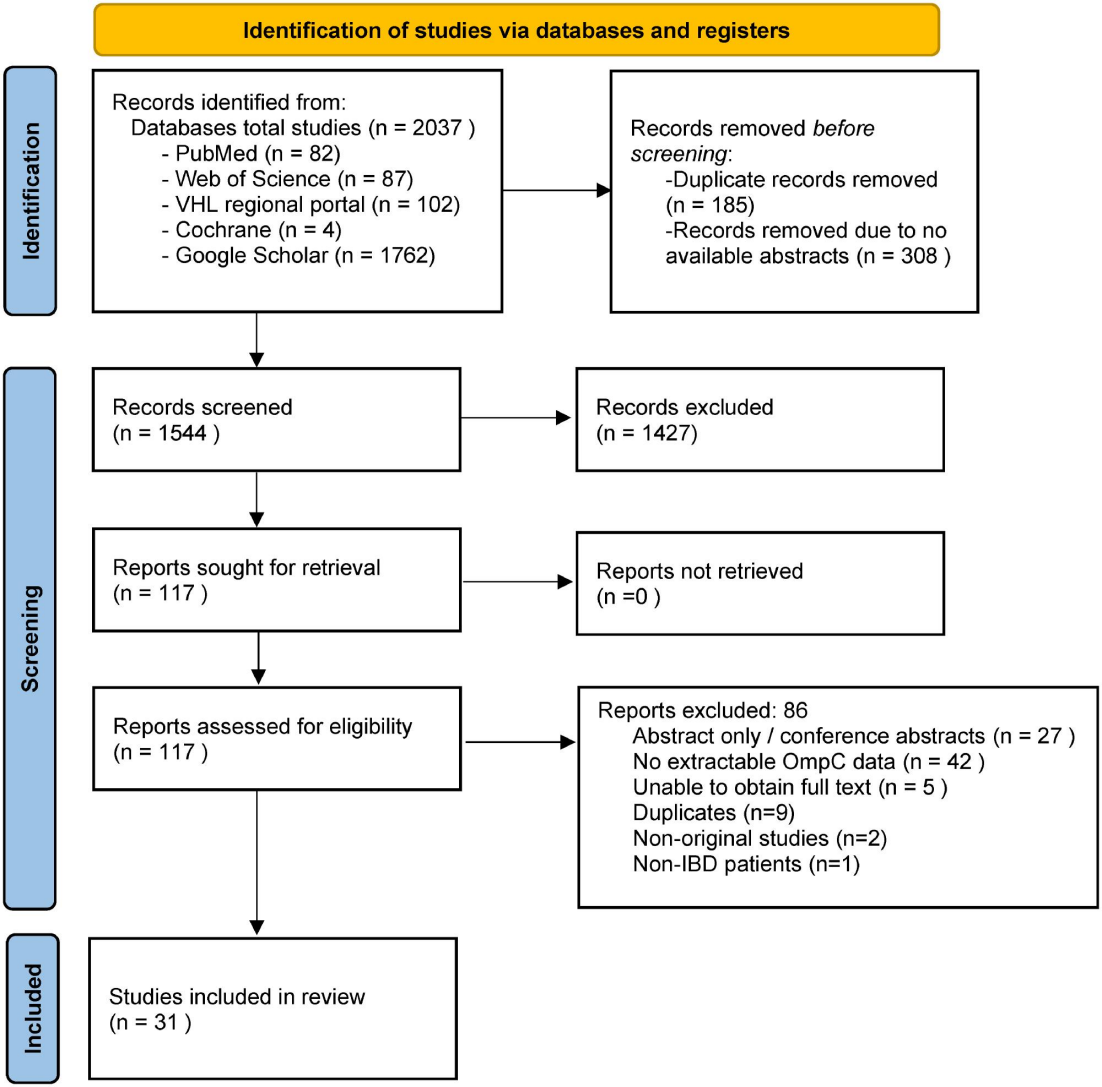
PRISMA 2020 flow diagram illustrating the study selection process for inclusion in the systematic review and meta-analysis of anti-OmpC antibodies in inflammatory bowel disease. *Source*: Page et al.^17^ This work is licensed under CC BY 4.0. To view a copy of this license, visit https://creativecommons.org/licenses/by/4.0/

The full electronic search strings and number of results per database are provided in Table S1. The study selection process is summarized in the PRISMA 2020 flow diagram (Figure 1).

### Eligibility criteria

Inclusion: Human studies reporting anti-OmpC positivity in CD and/or UC, with or without healthy controls. Studies with extractable data for prevalence, OR, sensitivity, or specificity.

Exclusion: Reviews, case reports, pediatric-only studies, animal models, or studies without extractable data.

### Data extraction and quality assessment

Methodological quality was assessed using the Newcastle–Ottawa Scale (NOS)^19^ for observational prevalence and prognostic studies. For the 12 diagnostic accuracy studies, we additionally applied the QUADAS-2 tool,^20^ which evaluates risk of bias and applicability concerns across 4 domains: patient selection, index test, reference standard, and flow/timing. Two reviewers independently performed the assessments, and disagreements were resolved by consensus. In addition to outcomes, we extracted study-level variables including population characteristics, study design, assay type and cutoff threshold, and year of publication. We also recorded whether diagnostic or prognostic endpoints were reported. Missing or unclear data were resolved by consensus among reviewers.

### Statistical analysis

Effect sizes: Odds ratios (OR) with 95% CIs using random-effects models.

Prevalence: Pooled using logit-transformed random-effects models.

Diagnostic accuracy: Sensitivity, specificity, DOR, and summary receiver operating characteristic curves estimated with bivariate random-effects models.^21^

Heterogeneity: Q test, I^2^, τ^2^.

Publication bias: Funnel plots, Egger’s regression, trim-and-fill.^22^

Subgroup analyses: By assay cutoff.

Sensitivity analyses: Primary analyses were restricted to studies using standardized ELISA cutoff thresholds with clearly defined numerical values. Studies with nonstandard or non-numeric cutoffs (e.g., quartile-based, ROC-derived, or relative thresholds such as >2 SD above controls) were excluded due to limited comparability across studies and potential misclassification bias. Sensitivity analyses including these studies were performed to assess robustness.

Post-test probability: Fagan nomograms.^23^

All analyses were performed using Comprehensive Meta-Analysis (CMA, version 4.0.000; Biostat, Englewood, NJ, USA)^24^ and R software (version 4.5.1; R Foundation for Statistical Computing, Vienna, Austria)^25^ with the *meta* package^26^ and *mada* package^27^. Prediction intervals are not reported by CMA when too few studies are included in a subgroup analysis. We did not perform a formal GRADE assessment. Certainty in the body of evidence was instead judged narratively, based on study quality assessments (NOS and QUADAS-2), the consistency of results across sensitivity analyses, and the robustness of pooled effect estimates.

## Results

### Study selection

A total of 2037 records were identified across databases. After screening and eligibility assessment, 31 studies fulfilled inclusion criteria and were analyzed (Figure 1).^3–16^,28-44 The key characteristics of the included studies are summarized in Table 1.

**Table 1:** Characteristics of included studies.

Study	Country	Design	Population (CD/UC/Control)	Assay type	Cutoff	Outcomes reported
**Wang et al.^11^**	China	Case–control observational study	26 CD, 34 UC, 30 healthy controls	indirect immunofluorescence	7.807 ng/mL	UC and CD sensitivity, specificity, prevalence, OR
**Kohoutova et al.^6^**	Czech Republic	Prospective study	86 CD, 25 UC, 45 healthy controls	ELISA	25 EU/mL	UC and CD sensitivity, specificity, prevalence, OR
**Devlin et al.^5^**	United States	Observational cohort study	732 CD, 220 unaffected relatives, 200 healthy controls	ELISA	EU/mL above reference range (not reported)	CD prevalence
**White et al.^43^**	United States	Prospective cohort study	334 UC/IBDU patients undergoing colectomy with IPAA	ELISA	>2 SD above the mean control titer	Backwash ileitis and pouchitis development (not enough studies for pooled data)
**Papadakis et al.^10^**	United States	Cross-sectional observational study	731 CD patients	ELISA	>2 SD above the mean control titer	CD prevalence
**Darwish et al.^12^**	Egypt	Cross-sectional study	45 UC, 45 controls	ELISA	13.8 EU/mL	UC Sensitivity, specificity, prevalence, OR, disease activity
**Bertin et al.^30^**	France	Prospective study	67 CD, 35 UC, 37 controls	ELISA	25 EU/mL	UC and CD Sensitivity, specificity, prevalence, OR, extra-intestinal manifestations, need for surgery, CD disease activity
**Ahmed et al.^28^**	United States	Retrospective cohort study	135 CD patients	ELISA	Prometheus’ diagnostic algorithm (not numerical values)	CD disease activity
**Mow et al.^9^**	United States	Observational cohort study	303 CD patients	ELISA	11.5 EU/mL	CD prevalence, complicated phenotype (B2/B3), disease location, need for surgery
**Kaur et al.^34^**	United States	Case-control study	1721 CD patients	ELISA	11.5 EU/mL	Disease location
**Bertha et al.^29^**	United States	Cross-sectional study	358 CD African American patients	ELISA	23 EU/mL	CD complicated phenotype (B2/B3), extra-intestinal manifestations, need for surgery, disease location
**Sura et al.^41^**	United States	Observational cohort study	117 indeterminate colitis → followed 1 yr (58 UC, 49 CD, 10 IC)	ELISA	16.5 EU/mL	UC and CD prevalence
**Lichtenstein et al.^37^**	United States and Canada	Cross-sectional study	593 CD patients	ELISA	Q4 was +ve	CD complicated phenotype (B2/B3)
**Ippoliti et al.^33^**	United States	Observational, cross-sectional analysis of an IBD database cohort	731 CD patients	ELISA	Quartile sum score 4-16 considered +ve	CD prevalence
**Plevy et al.^39^**	United States and Canada	Cross-sectional study	572 CD, 328 UC, 437 non-IBD GI controls, 183 healthy controls	ELISA	Q3 and Q4 were +ve	UC and CD Sensitivity, specificity, prevalence, OR
**Quezada et al.^40^**	United States	Cross-sectional, single-center study	47 CD patients	ELISA	No numerical cutoff	Age in relation to OmpC (not enough studies for pooled analysis)
**Mei et al.^7^**	United States	Observational, cross-sectional analysis	787 CD, 389 UC, 619 unaffected relatives, 216 healthy controls	ELISA	23 EU/mL	UC and CD sensitivity, specificity, prevalence, OR
**Michielan et al.^8^**	Italy	Retrospective study	60 CD, 86 unaffected first-degree relatives, 100 healthy controls	ELISA	25 EU/mL	CD sensitivity, specificity, prevalence, OR
**O’Donnell et al.^14^**	Ireland	Retrospective cohort with prospective serologic/genetic analysis	115 CD, 63 UC, 63 IAI, 63 healthy controls	ELISA	40 EU/mL	UC and CD sensitivity, specificity, prevalence, OR
**Papp et al.^38^**	Hungary	Multicenter cohort study	557 CD, 95 UC, 48 non-IBD GI controls, 100 healthy controls	ELISA	25 EU/mL	CD prevalence and complicated phenotype (B2/B3), disease location, need for surgery
**Ye et al.^44^**	China	Prospective observational study	130 CD, 120 UC, 80 healthy controls	ELISA	16 EU/mL	UC and CD sensitivity, specificity, prevalence, OR, CD complicated phenotype (B2/B3)
**Arnott et al.^3^**	United Kingdom (Scotland)	Cross-sectional observational study	142 CD patients	ELISA	Unknown (no numeric cutoff)	CD prevalence
**Choung et al.^4^**	United States	Case–control study using a prospective serum repository	100 CD, 522 non-IBD GI controls, 200 healthy controls	ELISA	11.5 EU/mL	CD prevalence, complicated phenotype (B2/B3)
**Hui et al.^32^**	United States	Prospective cohort study	28 IC undergoing colectomy with IPAA	ELISA	20.9 EU/mL	Acute and chronic pouchitis (specific population, not enough studies to run pool analysis)
**Wang et al.^42^**	China	Case–control study	71 CD, 41 UC, 31, healthy controls, 78 non-IBD GI controls	ELISA	>ROC-based threshold	UC and CD sensitivity, specificity, prevalence, OR
**Kristensen et al.^35^**	Norway	Prospective cohort (20-year follow up)	237 CD patients	ELISA	10.9 EU/mL	Need for surgery
**Kevans et al.^13^**	Canada	Retrospective cohort study	230 UC patients	ELISA	10.9 EU/mL (for 101 patients) and 16.4 EU/mL (for129 patients)	UC prevalence
**Petersen et al.^15^**	Denmark	Observational, cross-sectional case–control study	51 UC, 43 CD, 19 IPAA, 60 controls	ELISA	16.5 EU/mL	UC and CD Sensitivity, specificity, prevalence, OR, disease activity
**Elkadri et al.^31^**	Canada	Observational, cross-sectional serological study	391 CD, 207 UC, 62 healthy controls	ELISA	16.4 EU/mL	UC and CD Sensitivity, specificity, prevalence, OR
**Le et al.^36^**	United States	Prospective cohort study	261 UC, 17 CD	ELISA	>2 SD above the mean control titer	UC and CD prevalence
**O’Donnell et al.^16^**	Ireland	Cross-sectional, single-center study	179 CD patients	ELISA	40 EU/mL	CD prevalence, complicated phenotype (B2/B3), disease location, need for surgery

Some studies reported non-numeric cutoffs (eg, >2 SD above mean, quartile sum scores) as defined by the original authors.

Abbreviations: CD = Crohn’s disease; ELISA = enzyme-linked immunosorbent assay; EU/mL = enzyme units per milliliter; IBDU = inflammatory bowel disease unclassified; IC = indeterminate colitis; IPAA = ileal pouch–anal anastomosis; *N* = sample size; UC = ulcerative colitis; WB = Western blot.

### CD vs healthy controls

**Figure 2:**
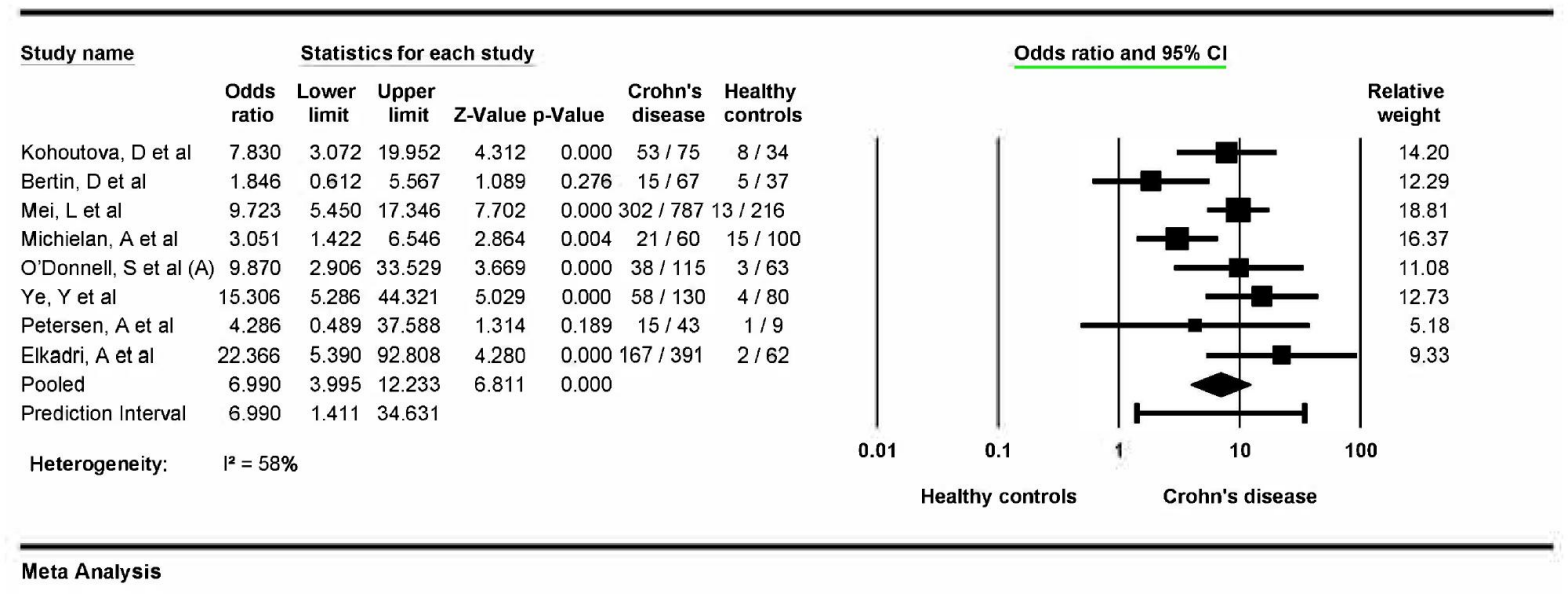
Forest plot of anti-OmpC antibody positivity in Crohn’s disease compared with healthy controls (primary analysis using standardized ELISA cutoffs).

Of 11 eligible CD-vs-control studies, 8 using standardized ELISA cutoffs, comprising 2342 participants, were included in the primary analysis, demonstrating a significant association (OR = 6.99, 95% CI 3.99-12.23, *P* < .001; I^2^ = 58%; prediction interval 1.41-34.63) (Figure 2).^6–8^^,^^11^^,^^14^^,^^15^^,^^30^^,^^31^^,^^39^^,^^42^^,^^44^ The 3 nonstandard cutoff studies were excluded.

Publication bias: Visual inspection of the funnel plot revealed no major asymmetry (Figure S1). Egger’s regression test confirmed this, with a non-significant intercept of 0.033 (*P* = .985).^22^ Duval and Tweedie’s trim-and-fill procedure did not impute any missing studies, and the adjusted pooled estimate remained unchanged at OR = 6.99, suggesting no publication bias.^22^

Subgroup analyses by anti-OmpC cutoff:

23-25 EU/mL (4 studies): OR = 4.85 (95% CI 2.27-10.34) (Figure S2).^6–8^^,^^30^

16-16.5 EU/mL (3 studies): OR = 14.53 (95% CI 6.58-32.11) (Figure S3).^15^^,^^31^^,^^44^

### Prevalence in CD

Twenty-one studies, comprising 5889 patients with CD, reported prevalence, with a pooled estimate of 36.9% (95% CI 33.8%-40.1%) (Figure S4).^3–11^^,^^14–16^^,^^30^^,^^31^^,^^33^^,^^36^^,^^38^^,^^39^^,^^41^^,^^42^^,^^44^ Heterogeneity was high (I^2^ = 79%). Egger’s intercept = –0.673 (*P* = .495).^22^ Trim-and-fill imputed 5 studies; adjusted prevalence = 39.3% (95% CI 35.9%-42.7%).^22^

Subgroups by anti-OmpC cutoff:

23-25 EU/mL: 38.8% (5 studies).^6–8^^,^^30^^,^^38^

16-16.5 EU/mL: 39.6% (4 studies).^15^^,^^31^^,^^41^^,^^44^

11.5 EU/mL: 34.7% (2 studies).^4^^,^^9^

40 EU/mL: 29.7% (2 studies).^14^^,^^16^

Non-numeric: 37.5% (7 studies).^3^^,^^5^^,^^10^^,^^33^^,^^36^^,^^39^^,^^42^

One outlier (Wang, J et al, IIF assay)^11^ reported 11.5% and was excluded.

Sensitivity analysis: 13 numeric cutoff studies: prevalence 36.6% (95% CI 31.6%-42.0%) (Figure S5).

### Diagnostic accuracy in CD

Across 11 studies (sensitivity analysis: *n* = 2337; specificity analysis: *n* = 1282), pooled sensitivity was 0.39 (95% CI 0.29-0.49; I^2^ = 80.9%).^6–8^^,^^11^^,^^14^^,^^15^^,^^30^^,^^31^^,^^39^^,^^42^^,^^44^ Forest plots with pooled and subgroup estimates by assay cutoff are shown (Figure S6).

Pooled specificity was 0.89 (95% CI 0.81-0.94; I^2^ = 79.0%), with subgroup analyses by cutoff displayed (Figure S7).

Bivariate model (numeric cutoffs)^21^: Sensitivity 0.40, Specificity 0.90, area under the curve (AUC) 0.76, DOR 6.99 (Figure S8).

Fagan nomogram^23^: Pre-test 50%; post-test 80.2% positive, 39.8% negative (Figure S9).

### UC vs healthy controls

**Figure 3:**
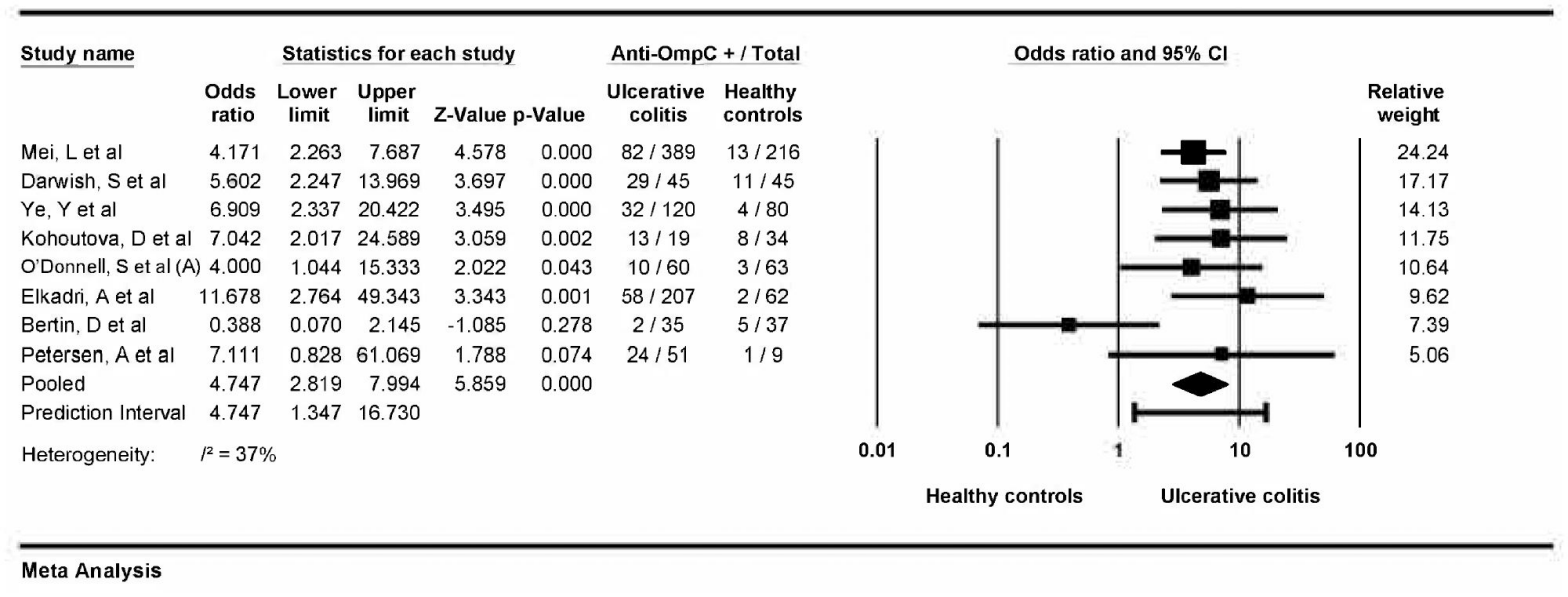
Forest plot of anti-OmpC antibody positivity in ulcerative colitis compared with healthy controls (primary analysis using standardized ELISA cutoffs).

Of 11 eligible UC-vs-control studies, 8 using standardized ELISA cutoffs, comprising 1543 participants, were included in the primary analysis, demonstrating a significant association (OR = 4.75, 95% CI 2.82-7.99, *P* < .001; I^2^ = 37%; prediction interval 1.35-16.73) (Figure 3).^6^^,^^7^^,^^11^^,^^12^^,^^14^^,^^15^^,^^30^^,^^31^^,^^39^^,^^42^^,^^44^ The 3 nonstandard cutoff studies were excluded.

Publication bias: Visual inspection of the funnel plot did not reveal major asymmetry (Figure S10). Egger’s regression test was non-significant (intercept = –0.26, *P* = .850).^22^ Duval and Tweedie’s trim-and-fill procedure imputed 2 potentially missing studies, producing an adjusted pooled OR of 4.18 (95% CI: 2.57-6.80), which remained statistically significant.^22^

Subgroup analyses by anti-OmpC cutoff:

23-25 EU/mL (3 studies): OR = 2.68 (95% CI 0.72-9.96) (Figure S11).^6^^,^^7^^,^^30^

16-16.5 EU/mL (3 studies): OR = 8.17 (95% CI 3.66-18.24) (Figure S12).^15^^,^^31^^,^^44^

### Prevalence in UC

Fourteen studies, comprising 1887 patients with UC, reported prevalence, with a pooled estimate of 26.8% (95% CI 20.8%-33.8%) (Figure S13).^6^^,^^7^^,^^11–15^^,^^30^^,^^31^^,^^36^^,^^39^^,^^41^^,^^42^^,^^44^ Heterogeneity was high (I^2^ = 86%). Egger’s intercept = 1.26 (*P* = .441).^22^ Trim-and-fill imputed 2 studies; adjusted prevalence = 29.1% (95% CI 22.5%-36.6%).^22^

Subgroups by anti-OmpC cutoff:

23-25 EU/mL: 25.8% (3 studies).^6^^,^^7^^,^^30^

16-16.5 EU/mL: 31.2% (4 studies).^15^^,^^31^^,^^41^^,^^44^

40 EU/mL: 16.7% (1 study).^14^

13.8 EU/mL: 64.4% (1 study).^12^

Mixed cutoff: 17.8% (1 study).^13^

Non-numeric: 21.1% (3 studies).^36^^,^^39^^,^^42^

One outlier (IIF assay)^11^ reported 1.4% and was excluded.

Sensitivity analysis: 10 numeric cutoff studies: prevalence 29.8% (95% CI 21.7%-39.4%) (Figure S14).

### Diagnostic accuracy in UC

Across 11 studies (sensitivity analysis: *n* = 1329; specificity analysis: *n* = 1227), pooled sensitivity was 0.30 (95% CI 0.22-0.39; I^2^ = 86.6%). Forest plots with pooled and subgroup estimates by assay cutoff are shown (Figure S15).^6^^,^^7^^,^^11^^,^^12^^,^^14^^,^^15^^,^^30^^,^^31^^,^^39^^,^^42^^,^^44^

Pooled specificity was 0.88 (95% CI 0.81-0.93; I^2^ = 80.5%), with subgroup analyses by cutoff displayed (Figure S16).

Bivariate model (numeric cutoffs)^21^: Sensitivity 0.34, specificity 0.89, AUC 0.77, DOR 4.64 (Figure S17).

Fagan nomogram^23^: Pre-test 50%; post-test 77.1% positive, 42.2% negative (Figure S18).

Figure S19 presents the combined summary ROC curves for anti-OmpC in both CD and UC derived from the bivariate model.

### Clinical associations

#### Complicated CD

**Figure 4:**
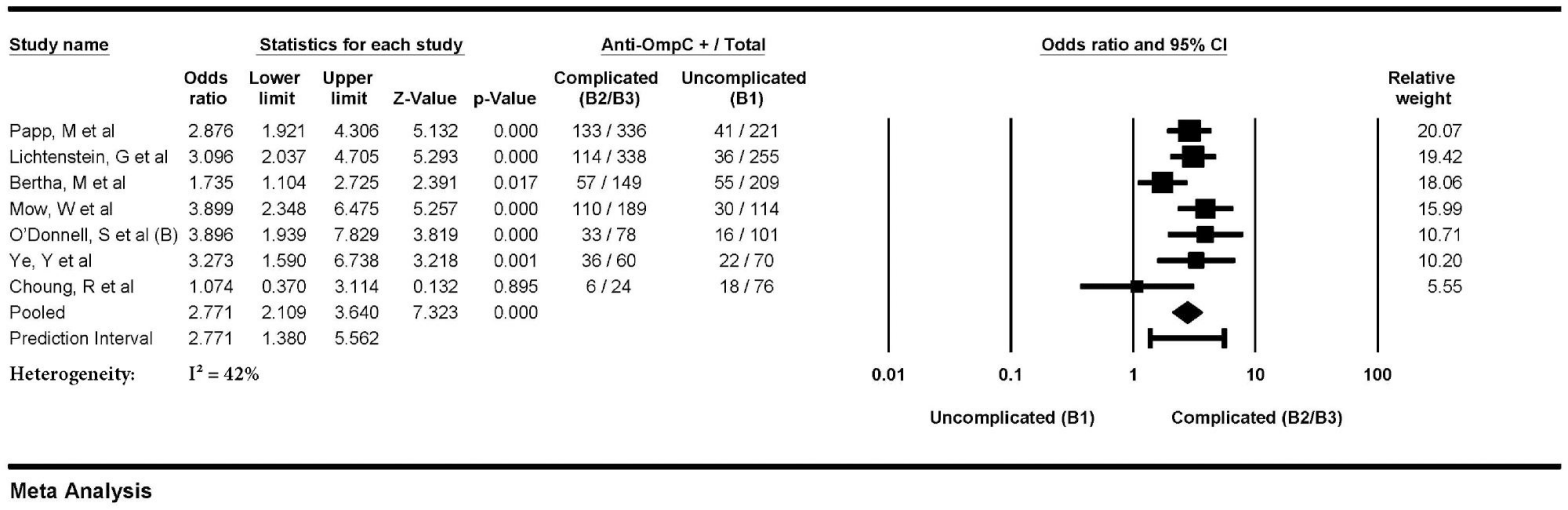
Forest plot showing the association between anti-OmpC antibody positivity and complicated Crohn’s disease behavior (stricturing/penetrating, B2/B3 vs inflammatory, B1).

Seven studies, comprising 2220 patients with CD, evaluated the association between OmpC positivity and disease behavior.^4^^,^^9^^,^^16^^,^^29^^,^^37^^,^^38^^,^^44^ The pooled analysis demonstrated a significant association with complicated CD (stricturing or penetrating, B2/B3 vs inflammatory, B1), with an OR of 2.77 (95% CI: 2.11-3.64, *P* < .001) (Figure 4). This association remained robust in sensitivity analyses excluding studies with nonstandard cutoffs, and the prediction interval (OR range 1.38-5.56) supported generalizability across populations.

#### Need for surgery

**Figure 5:**
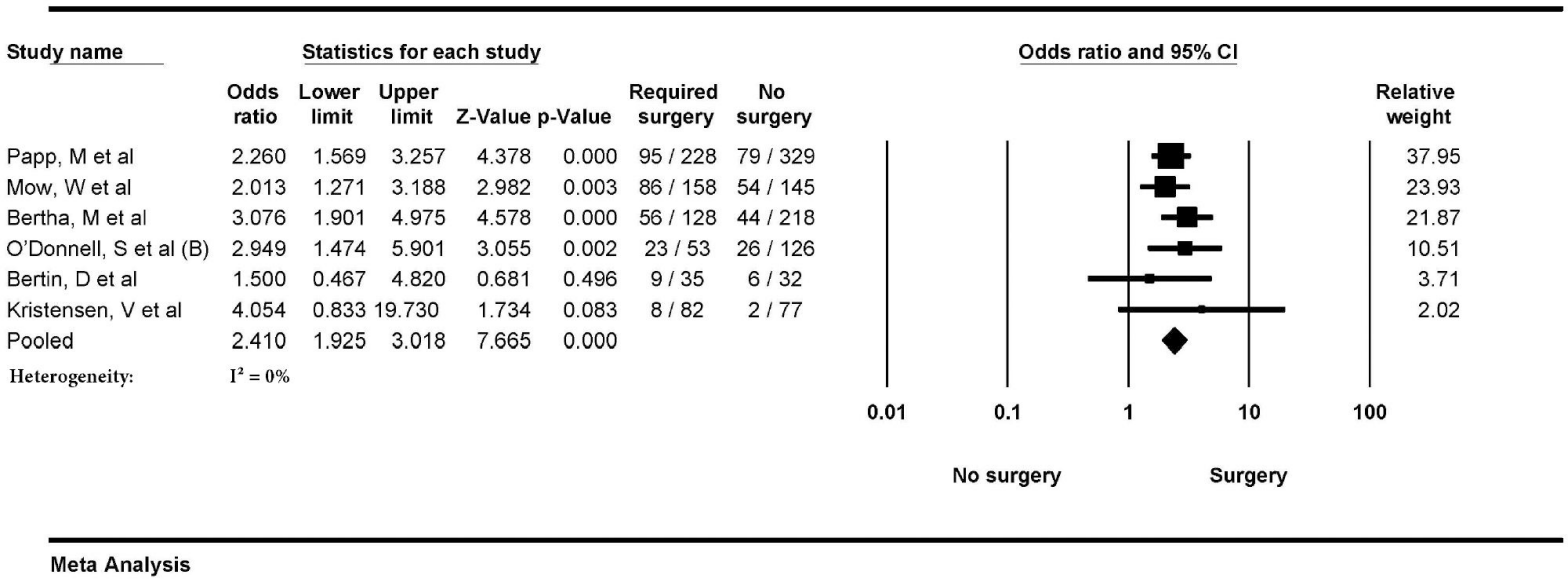
Forest plot showing the association between anti-OmpC antibody positivity and the need for surgery in Crohn’s disease.

Six studies, comprising 1701 patients with CD, reported on surgical outcomes.^9^^,^^16^^,^^29^^,^^30^^,^^35^^,^^38^ OmpC-positive patients had significantly greater odds of requiring surgery compared with OmpC-negative patients (OR = 2.41, 95% CI: 1.93-3.02, *P* < .001) (Figure 5). Heterogeneity was negligible (I^2^ = 0%), strengthening confidence in the consistency of this association.

#### Other associations

Location: small bowel OR = 1.08 (95% CI: 0.82-1.43, *P* = .578),^9^^,^^16^^,^^29^^,^^38^ indicating no significant association; However, perianal disease showed a significant association, with an OR of 1.60 (95% CI: 1.31-1.96, *P* < .001).^29^^,^^34^ Due to the limited number of studies, heterogeneity and publication bias were not formally assessed, in line with recommended guidelines.

Extra-intestinal manifestations: OR = 1.17 (95% CI: 0.74-1.86, *P* = .501), indicating no significant association.^29^^,^^30^

#### Quality assessment

The NOS^19^ results are summarized in Table S2 and visualized in Figure S20. In addition, QUADAS-2 assessment of the 12 diagnostic accuracy studies demonstrated generally low risk of bias across domains, though concerns were noted for patient selection (case–control design in several studies) and reference standard (lack of uniform gold standard). Applicability concerns were judged low overall. The domain-level assessments are shown in a traffic light plot (Figure S21). Overall certainty of the evidence was considered moderate to high for CD outcomes and moderate for UC outcomes, reflecting low-to-moderate risk of bias, consistent findings across sensitivity analyses, and robust effect estimates despite heterogeneity.

## Discussion

This systematic review and meta-analysis provides the most comprehensive synthesis to date of anti-OmpC antibodies in IBD. Our findings confirm that anti-OmpC positivity is strongly associated with both CD and UC, with consistently high specificity but modest sensitivity. Importantly, in CD, OmpC positivity was significantly linked to complicated disease behavior and the need for surgery, highlighting a consistent association with adverse disease outcomes. These observations underscore that the clinical utility of OmpC lies less in primary diagnosis and more in characterizing patients with more severe disease phenotypes.

### Clinical relevance and guideline context

From a clinical perspective, these results refine the role of serological testing in IBD. Current international guidelines emphasize a combination of clinical, endoscopic, radiological, and serological parameters for diagnosis and prognostication.^45^ While ASCA and pANCA remain the most established antibodies,^1^^,^^2^ our synthesis highlights that anti-OmpC contributes complementary information. Its high specificity (0.89 in CD, 0.88 in UC) may provide reassurance in borderline diagnostic cases, especially when used in conjunction with other antibodies. More importantly, in CD, OmpC positivity identifies a subgroup of patients with increased odds of developing stricturing or penetrating complications and of ultimately requiring surgery. These results underscore the potential of OmpC as a prognostic marker that could be integrated into evidence-based risk stratification strategies.

### Prognostic strength in CD

The strongest signal observed for OmpC relates to its association with disease severity rather than diagnostic performance. Our pooled analyses demonstrated that OmpC positivity nearly tripled the odds of complicated CD (OR = 2.77) and more than doubled the odds of surgical intervention (OR = 2.41). These consistent associations, with low heterogeneity for surgical outcomes, emphasize that OmpC may reflect a consistent association with more severe disease phenotypes across populations. These findings suggest a potential relationship between serological response and disease severity; however, prospective studies are required before clinical application can be considered.

### Comparison with established antibodies

Compared with existing serological markers, anti-OmpC demonstrates unique features. ASCA is more sensitive but less prognostic, while pANCA is primarily relevant to UC.^1^^,^^2^ Anti-OmpC, in contrast, shows clinical utility across both IBD subtypes, with added prognostic power in CD. CBir1 (anti-flagellin) has also been linked to complicated Crohn’s phenotypes,^10^^,^^46^ yet is not as consistently validated as OmpC. Taken together, these data support a complementary, rather than competitive, role for OmpC within serological panels. When integrated into multi-marker algorithms alongside ASCA, pANCA, and CBir1, OmpC may enhance predictive accuracy for disease progression and treatment response.^47^^,^^48^ Such approaches have already been explored in retrospective studies and are increasingly relevant in the era of personalized medicine.

### UC: Supportive but limited evidence

In UC, the available evidence is smaller and more heterogeneous. Our pooled OR of 4.75 indicates a significant association with disease, but sensitivity was modest (0.30), and prevalence varied widely across studies. While these findings support an association between anti-OmpC antibodies and UC, the overall evidence remains limited and heterogeneous, and the clinical utility of this marker in UC is uncertain. Further prospective studies with standardized assays are needed to clarify its role. Given the heterogeneity, we interpret the UC findings as supportive but exploratory, warranting further validation in prospective cohorts.

### Strengths and limitations

Major strengths of this review include adherence to PRISMA guidelines,^17^ protocol registration,^18^ comprehensive literature coverage, and the use of robust statistical models, including subgroup, sensitivity, and publication bias analyses.^22^ The inclusion of Fagan nomograms further contextualizes the results for clinical practice.^23^ However, several limitations warrant consideration. Assay heterogeneity, particularly in cutoff thresholds, contributed to variability in prevalence estimates. UC analyses were limited by smaller sample sizes and broader confidence intervals. The NOS identified moderate to high study quality overall,^19^ but several included studies were cross-sectional. Despite these limitations, sensitivity analyses confirmed the robustness of the findings.

### Future directions

Looking forward, prospective longitudinal studies with standardized ELISA cutoffs will be critical to validating the prognostic role of OmpC. The integration of OmpC into multi-marker serological panels represents a particularly promising direction. Multi-marker approaches combining ASCA, pANCA, OmpC, and CBir1 may enable early prediction of complicated disease trajectories, surgical need, or treatment response.^46–48^ Ultimately, such biomarker-based algorithms may help bridge the gap between diagnosis and precision medicine in IBD.

## Conclusion

Taken together, this meta-analysis demonstrates that anti-OmpC is a highly specific serological marker with consistent associations with disease severity in CD. While not suitable as a standalone diagnostic tool, OmpC positivity is associated with increased odds of complications and surgery. In UC, although an association with disease was observed, the evidence is more limited and heterogeneous, and the clinical utility of OmpC remains uncertain. Incorporating OmpC into multi-marker panels could enhance risk stratification and inform future research on risk stratification approaches.^47^^,^^48^ These findings highlight the need for standardized assays and prospective validation, with the ultimate goal of integrating OmpC into precision-medicine approaches in IBD.

## Supplementary Material

otag056_Supplementary_Data

## Data Availability

All data underlying this systematic review and meta-analysis are available within the article and its Supplementary Material. Extracted datasets from included studies can be obtained from the corresponding author upon reasonable request.
