# Supplementary material for: Anti-OmpC antibodies in Crohn’s disease and ulcerative colitis: evidence from a systematic review and meta-analysis
Source: Crohns Colitis 360. 2026 Jun 12;8(2):otag056. doi: 10.1093/crocol/otag056 (PMC13312123; doi:10.1093/crocol/otag056)
Supplement: otag056_Supplementary_Data [file otag056_supplementary_data.zip › Supplementary material file.docx]

**Supplementary Materials**

**Supplemental Tables**

Supplementary Table S1. Electronic search strategies and number of records retrieved per database.

Supplementary Table S2. Newcastle–Ottawa Scale quality assessment.

**Supplemental Figures**

Supplementary Figure S1. Funnel plot for Crohn’s disease vs healthy controls (8 studies with numeric cutoffs).

Supplementary Figure S2. Forest plot for Crohn’s disease, anti-OmpC cutoff 23–25 EU/mL.

Supplementary Figure S3. Forest plot for Crohn’s disease, anti-OmpC cutoff 16–16.5 EU/mL.

Supplementary Figure S4. Forest plot of pooled prevalence of anti-OmpC positivity in Crohn’s disease (all studies).

Supplementary Figure S5. Forest plot of pooled prevalence of anti-OmpC positivity in Crohn’s disease (numeric cutoff studies only).

Supplementary Figure S6. Forest plot of pooled sensitivity of anti-OmpC antibodies in Crohn’s disease (11 studies with subgroup analyses).

Supplementary Figure S7. Forest plot of pooled specificity of anti-OmpC antibodies in Crohn’s disease (11 studies with subgroup analyses).

Supplementary Figure S8. Summary receiver operating characteristic (SROC) curve for anti-OmpC in Crohn’s disease (8 studies with numeric cutoffs).

Supplementary Figure S9. Fagan nomogram for Crohn’s disease showing pre-test and post-test probabilities of OmpC positivity.

Supplementary Figure S10. Funnel plot for ulcerative colitis vs healthy controls (8 studies with numeric cutoffs).

Supplementary Figure S11. Forest plot for ulcerative colitis, anti-OmpC cutoff 23–25 EU/mL.

Supplementary Figure S12. Forest plot for ulcerative colitis, anti-OmpC cutoff 16–16.5 EU/mL.

Supplementary Figure S13. Forest plot of pooled prevalence of anti-OmpC positivity in ulcerative colitis (all studies).

Supplementary Figure S14. Forest plot of pooled prevalence of anti-OmpC positivity in ulcerative colitis (numeric cutoff studies only).

Supplementary Figure S15. Forest plot of pooled sensitivity of anti-OmpC antibodies in ulcerative colitis (11 studies with subgroup analyses).

Supplementary Figure S16. Forest plot of pooled specificity of anti-OmpC antibodies in ulcerative colitis (11 studies with subgroup analyses).

Supplementary Figure S17. Summary receiver operating characteristic (SROC) curve for anti-OmpC in ulcerative colitis (numeric cutoff studies).

Supplementary Figure S18. Fagan nomogram for ulcerative colitis showing pre-test and post-test probabilities of OmpC positivity.

Supplementary Figure S19. Combined summary receiver operating characteristic (SROC) curve for anti-OmpC in Crohn’s disease and ulcerative colitis, based on the bivariate diagnostic model.

Supplementary Figure S20. Newcastle–Ottawa Scale (NOS) quality assessment of included studies.

Supplementary Figure S21. QUADAS-2 traffic light plot summarizing risk of bias and applicability concerns across diagnostic accuracy studies.
