# Supplementary material for: Anti-OmpC antibodies in Crohn’s disease and ulcerative colitis: evidence from a systematic review and meta-analysis
Source: Crohns Colitis 360. 2026 Jun 12;8(2):otag056. doi: 10.1093/crocol/otag056 (PMC13312123; doi:10.1093/crocol/otag056)
Supplement: otag056_Supplementary_Data [file otag056_supplementary_data.zip › Table S1. Full electronic search.docx]

# Table S1. Full electronic search strategies and results before deduplication

| **Database** | **Search date** | **Search string (verbatim)** | **Results before deduplication** |
| --- | --- | --- | --- |
| PubMed | 3 May 2025 | ((Inflammatory Bowel Diseases) OR (IBD) OR (Ulcerative Colitis) OR (Crohn’s disease)) AND ((anti-OmpC) OR (E. coli outer membrane porin C antibodies) OR (outer membrane porin C antibodies)) | 82 |
| VHL Regional Portal | 4 May 2025 | ((Inflammatory Bowel Diseases) OR (IBD) OR (Ulcerative Colitis) OR (Crohn’s disease)) AND ((anti-OmpC) OR (E. coli outer membrane porin C antibodies) OR (outer membrane porin C antibodies)) | 102 |
| Web of Science | 4 May 2025 | ((Inflammatory Bowel Diseases) OR (IBD) OR (Ulcerative Colitis) OR (Crohn’s disease)) AND ((anti-OmpC) OR (E. coli outer membrane porin C antibodies) OR (outer membrane porin C antibodies)) | 87 |
| Cochrane Library | 4 May 2025 | ((Inflammatory Bowel Diseases) OR (IBD) OR (Ulcerative Colitis) OR (Crohn’s disease)) AND ((anti-OmpC) OR (E. coli outer membrane porin C antibodies) OR (outer membrane porin C antibodies)) | 4 trials |
| Google Scholar | 5 May 2025 | Inflammatory bowel disease AND anti-OmpC | 1,762 (302 from 2020–2025; 392 from 2015–2019; 583 from 2010–2014; 479 from 2000–2009; 6 prior to 2000) |

Footnote: Results are reported as retrieved before deduplication. After removal of 185 duplicates and 308 records with no available abstracts, 1,544 records were screened.
