# Supplementary material for: Anti-OmpC antibodies in Crohn’s disease and ulcerative colitis: evidence from a systematic review and meta-analysis
Source: Crohns Colitis 360. 2026 Jun 12;8(2):otag056. doi: 10.1093/crocol/otag056 (PMC13312123; doi:10.1093/crocol/otag056)
Supplement: otag056_Supplementary_Data [file otag056_supplementary_data.zip › Table S2. NOS quality assessment..docx]

Table S2. Newcastle–Ottawa Scale (NOS) Quality Assessment of Included Studies

| Author (Year) | NOS Score (0–9) |
| --- | --- |
| Kristensen, V et al (2020) | 9 |
| Ye, Y et al (2019) | 9 |
| White, E et al (2010) | 9 |
| Papp, M et al (2008) | 9 |
| Elkadri, A et al (2013) | 9 |
| Kevans, D et al (2015) | 9 |
| Plevy, S et al (2013) | 8 |
| O’Donnell, S et al (A) (2013) | 8 |
| Michielan, A et al (2013) | 8 |
| Mei, L et al (2006) | 8 |
| Kohoutova, D et al (2014) | 8 |
| Choung, R et al (2016) | 8 |
| Kaur, M et al (2016) | 8 |
| Papadakis, K et al (2007) | 8 |
| Le, Q et al (2013) | 8 |
| Lichtenstein, G et al (2011) | 7 |
| Wang, Z et al (2017) | 7 |
| Hui, T et al (2005) | 7 |
| Petersen, A et al (2012) | 7 |
| Ahmed, Z et al (2020) | 7 |
| Bertin, D et al (2013) | 7 |
| Arnott, I et al (2004) | 7 |
| O’Donnell, S et al (B) (2013) | 7 |
| Wang, J et al (2018) | 6 |
| Ippoliti, A et al (2010) | 6 |
| Sura, S et al (2014) | 6 |
| Bertha, M et al (2018) | 6 |
| Mow, W et al (2004) | 6 |
| Devlin, S et al (2007) | 6 |
| Quezada, S et al (2015) | 6 |
| Darwish, S et al (2022) | 5 |

NOS = Newcastle–Ottawa Scale. Scores range from 0 (lowest quality) to 9 (highest quality).
