# Supplementary figures and images for: Anti-OmpC antibodies in Crohn’s disease and ulcerative colitis: evidence from a systematic review and meta-analysis
Source: Crohns Colitis 360. 2026 Jun 12;8(2):otag056. doi: 10.1093/crocol/otag056 (PMC13312123; doi:10.1093/crocol/otag056)

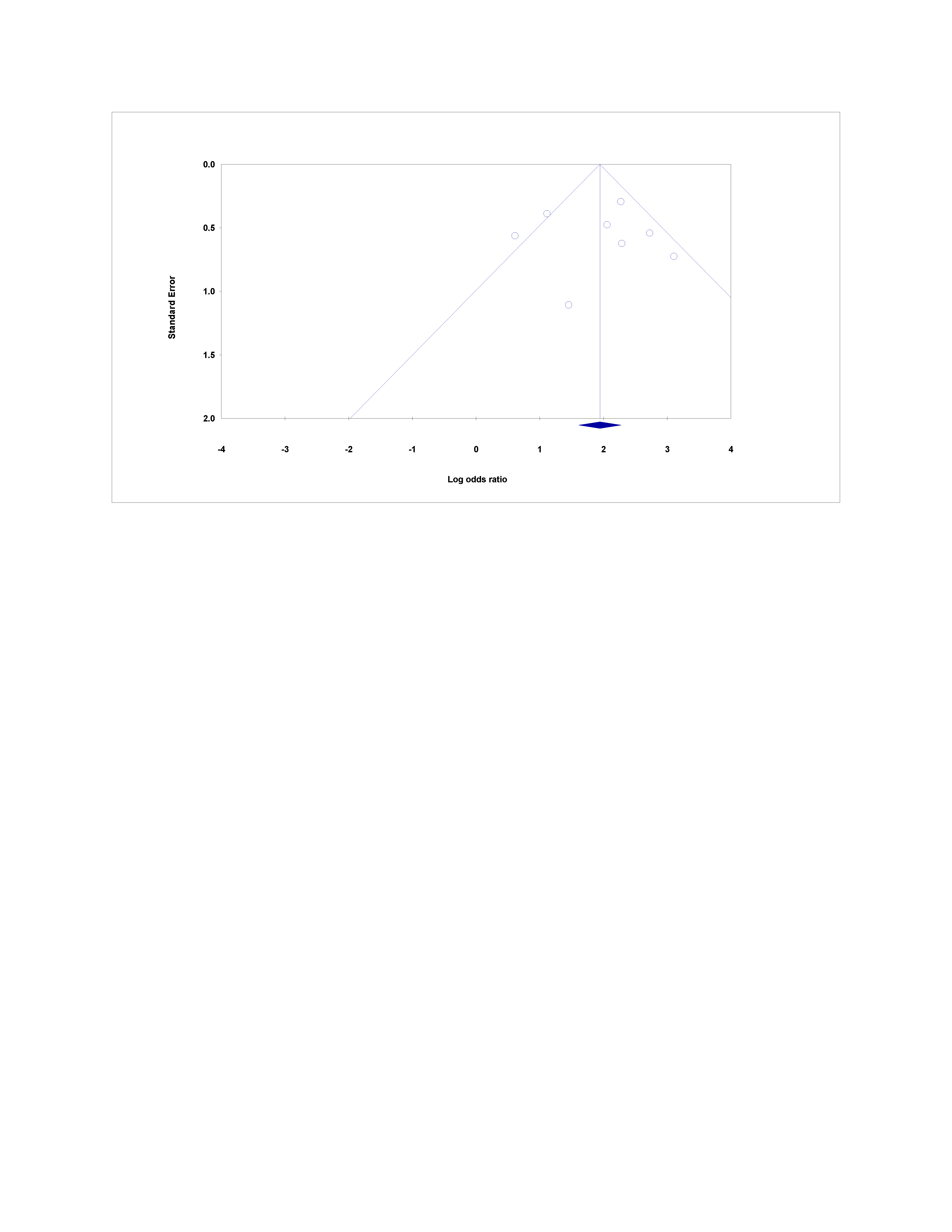

Supplement: otag056_Supplementary_Data [file otag056_supplementary_data.zip › S1. Funnel plot for main analysis (8 studies CD w OmpC).tif]

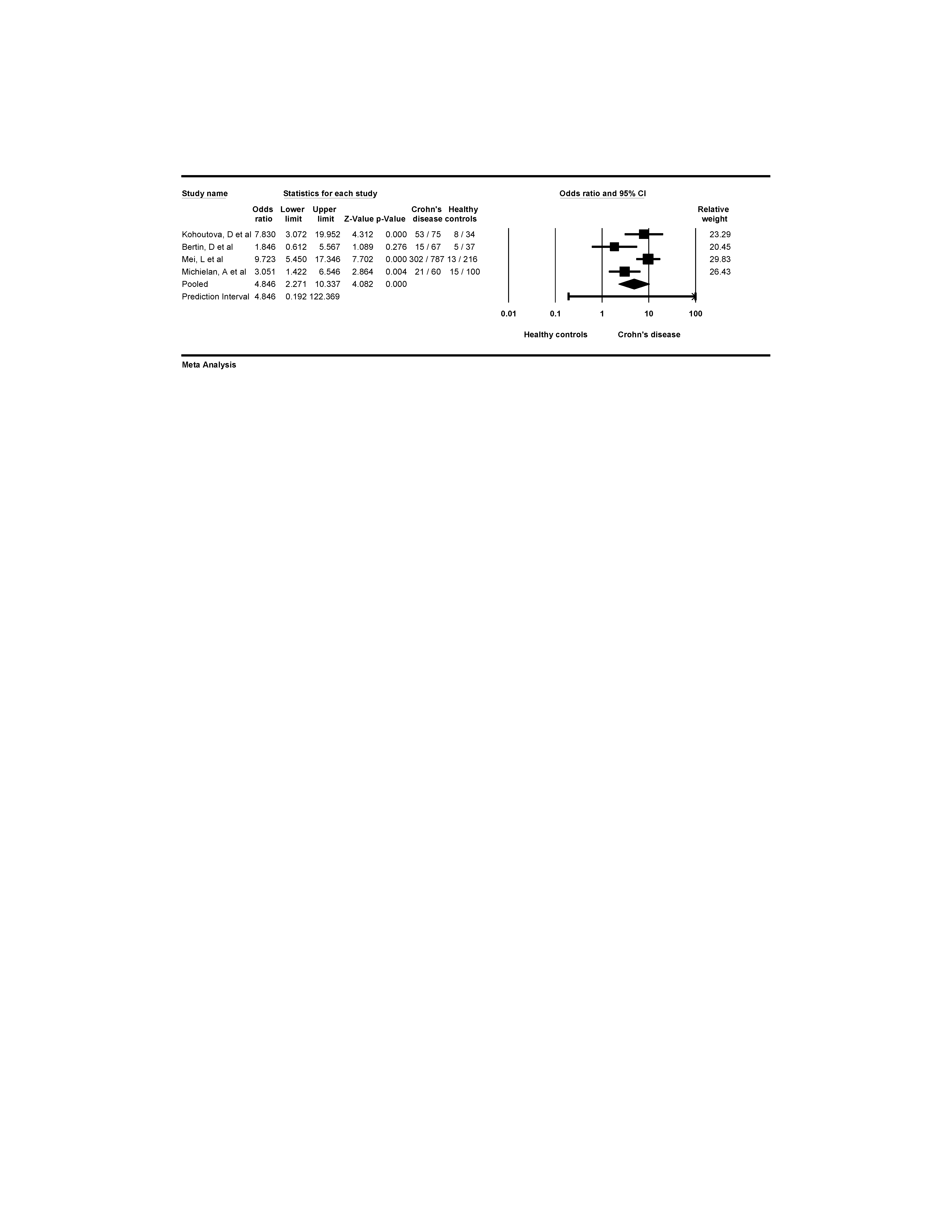

Supplement: otag056_Supplementary_Data [file otag056_supplementary_data.zip › S2. Forest plot (CD with OmpC, 23-25 cutoff).tif]

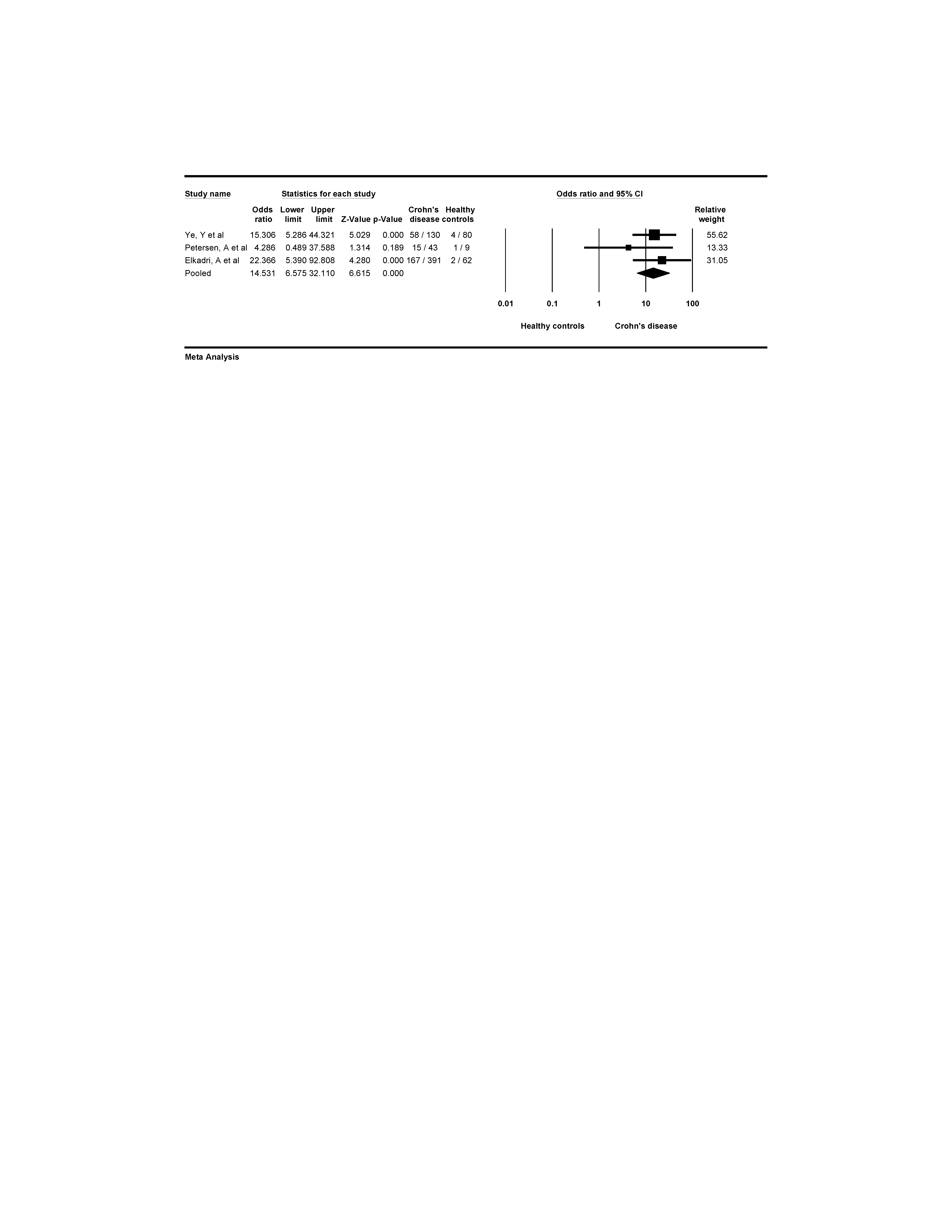

Supplement: otag056_Supplementary_Data [file otag056_supplementary_data.zip › S3. Forest plot (CD with OmpC, 16-16.5 cut off).tif]

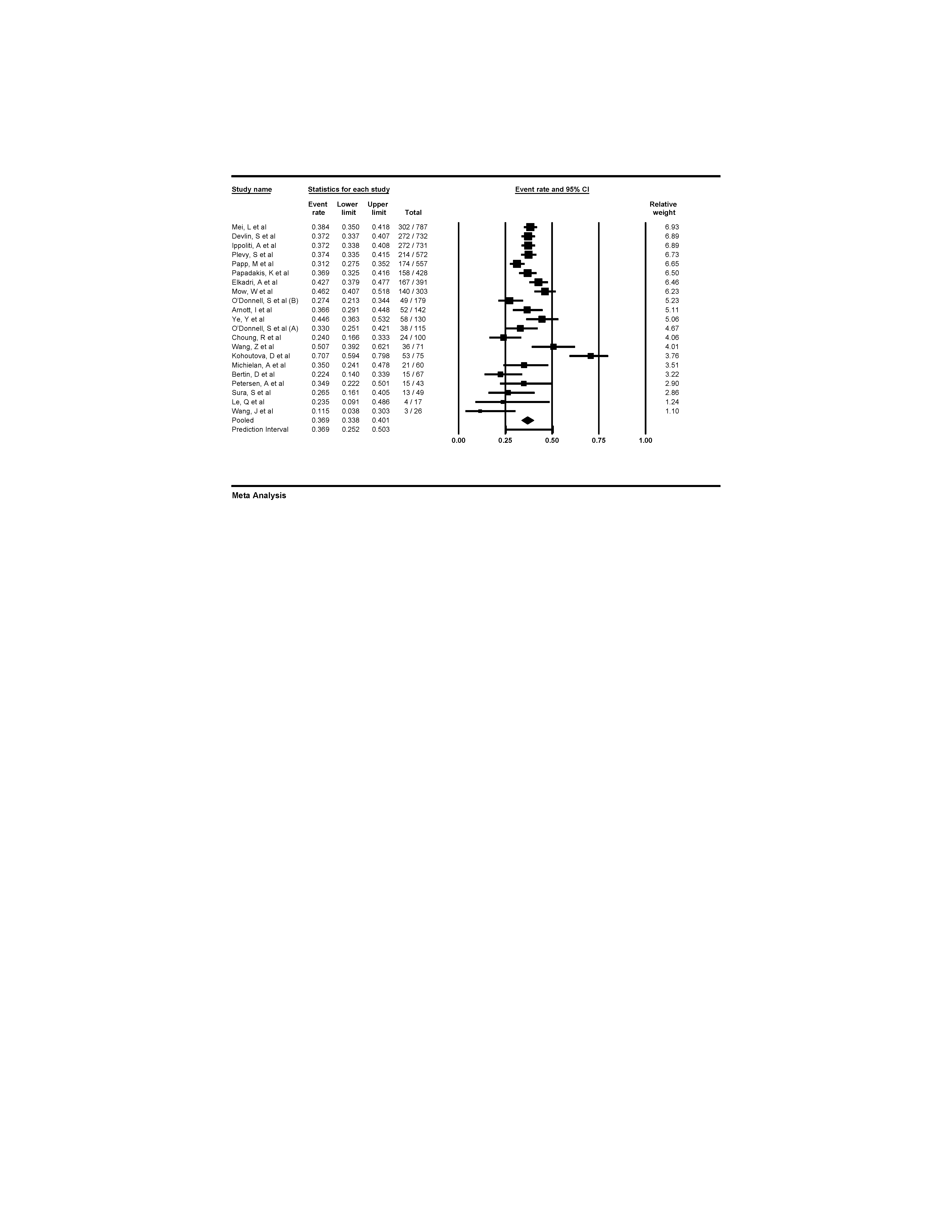

Supplement: otag056_Supplementary_Data [file otag056_supplementary_data.zip › S4. CD prevalence forest plot (All studies).tif]

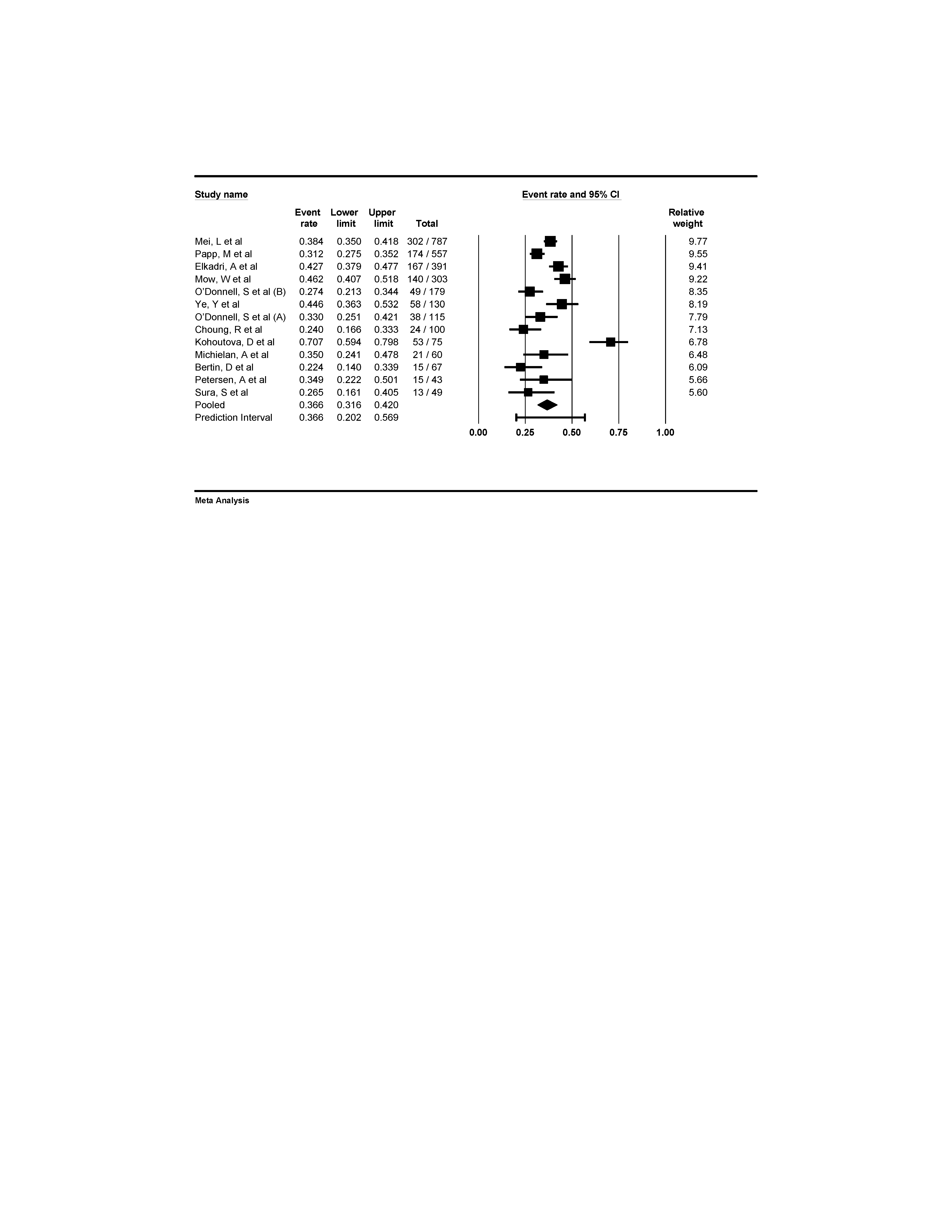

Supplement: otag056_Supplementary_Data [file otag056_supplementary_data.zip › S5. CD prevalence Forest plot (numeric cut off studies, 13).tif]

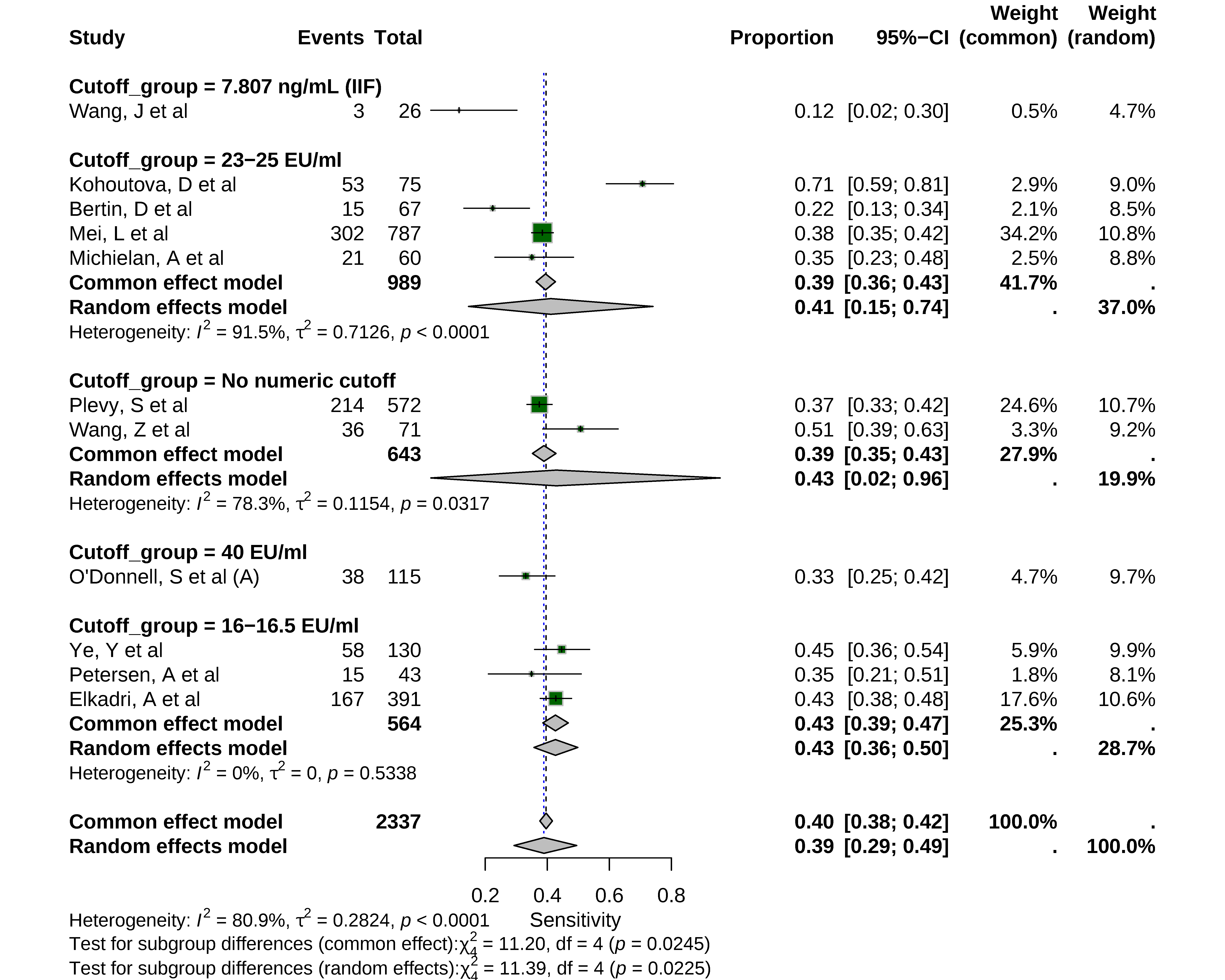

Supplement: otag056_Supplementary_Data [file otag056_supplementary_data.zip › S6. Pooled CD sensitivity (subgroups of 11 studies).tif]

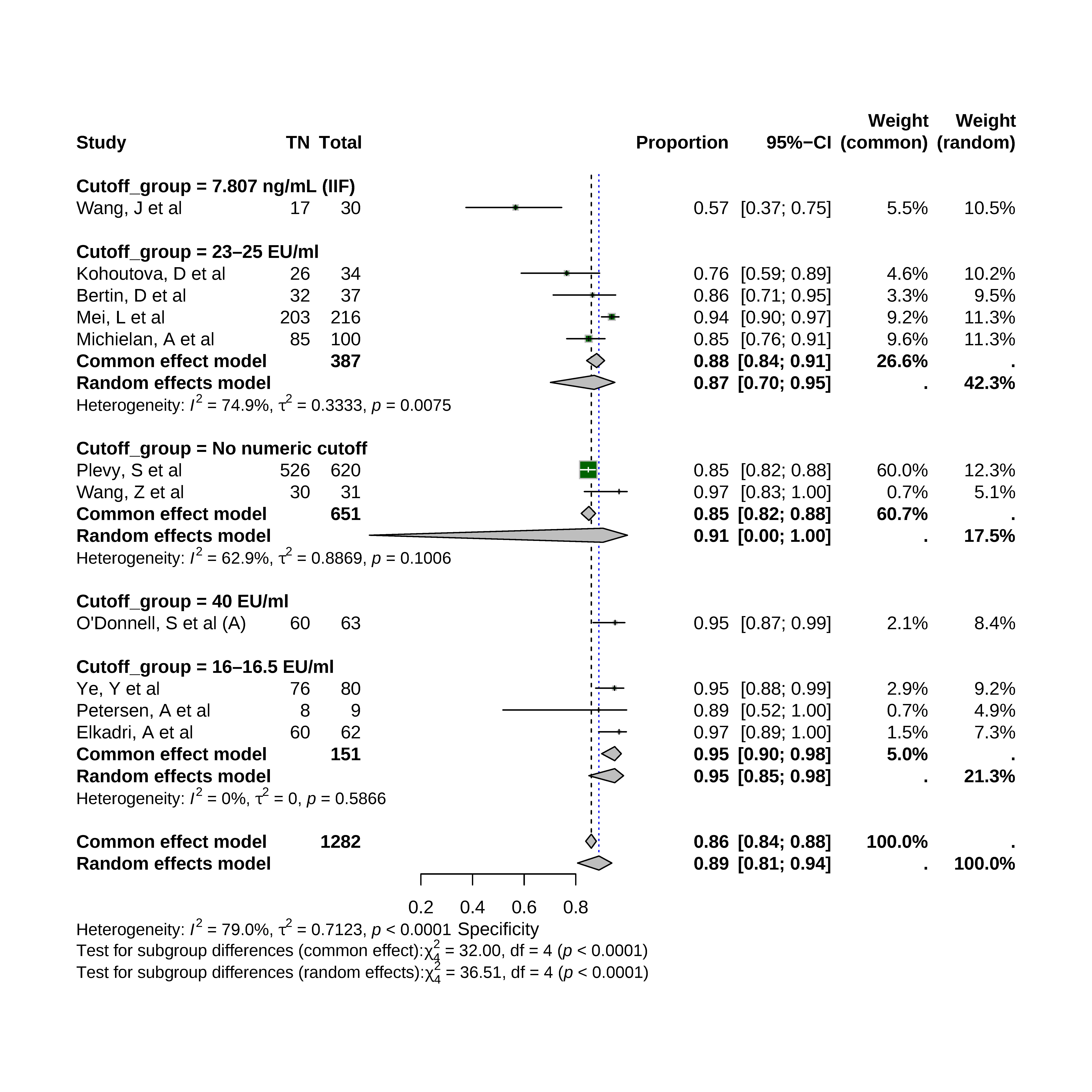

Supplement: otag056_Supplementary_Data [file otag056_supplementary_data.zip › S7. Pooled CD specificity (subgroup of 11 studies).tif]

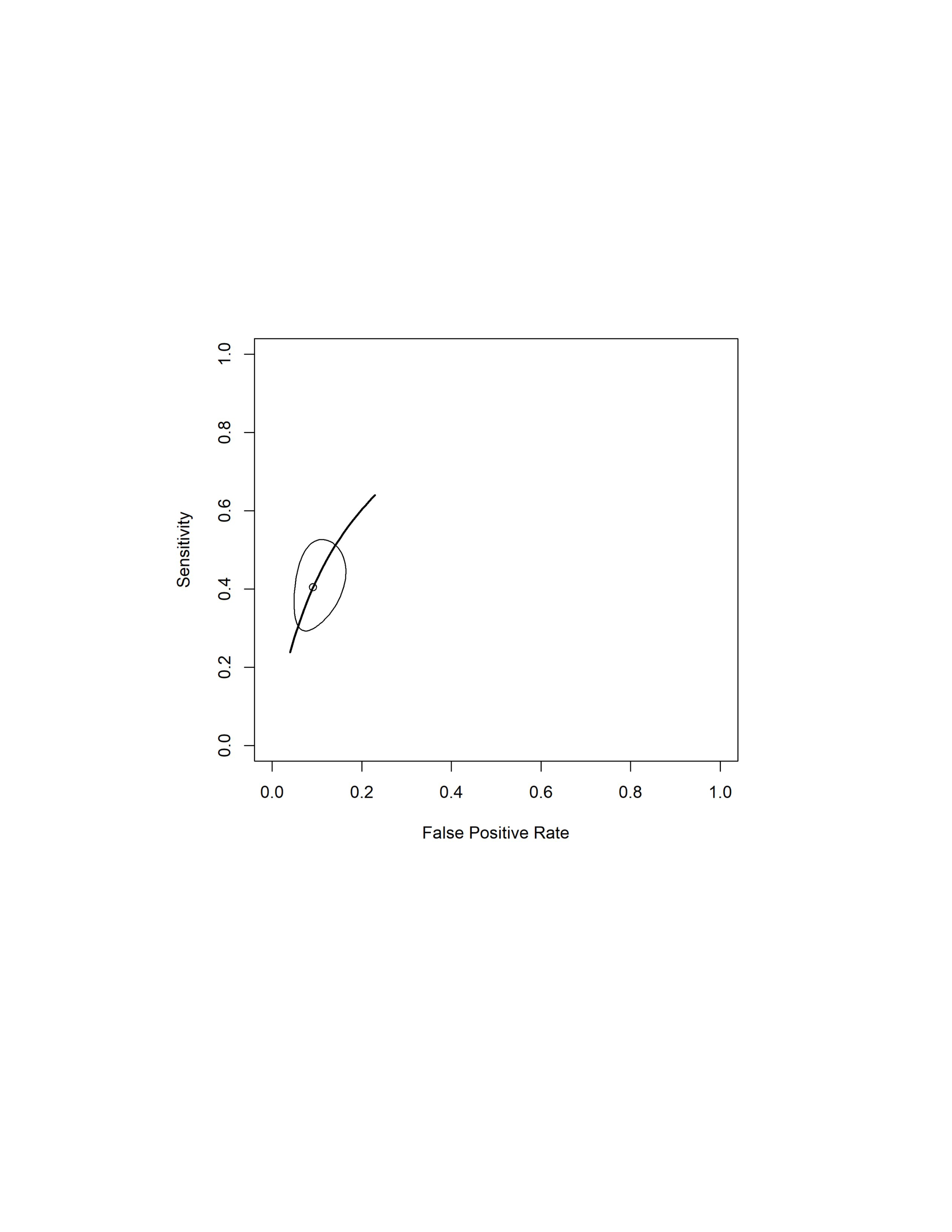

Supplement: otag056_Supplementary_Data [file otag056_supplementary_data.zip › S8. CD SROC (numeric 8 studies).tif]

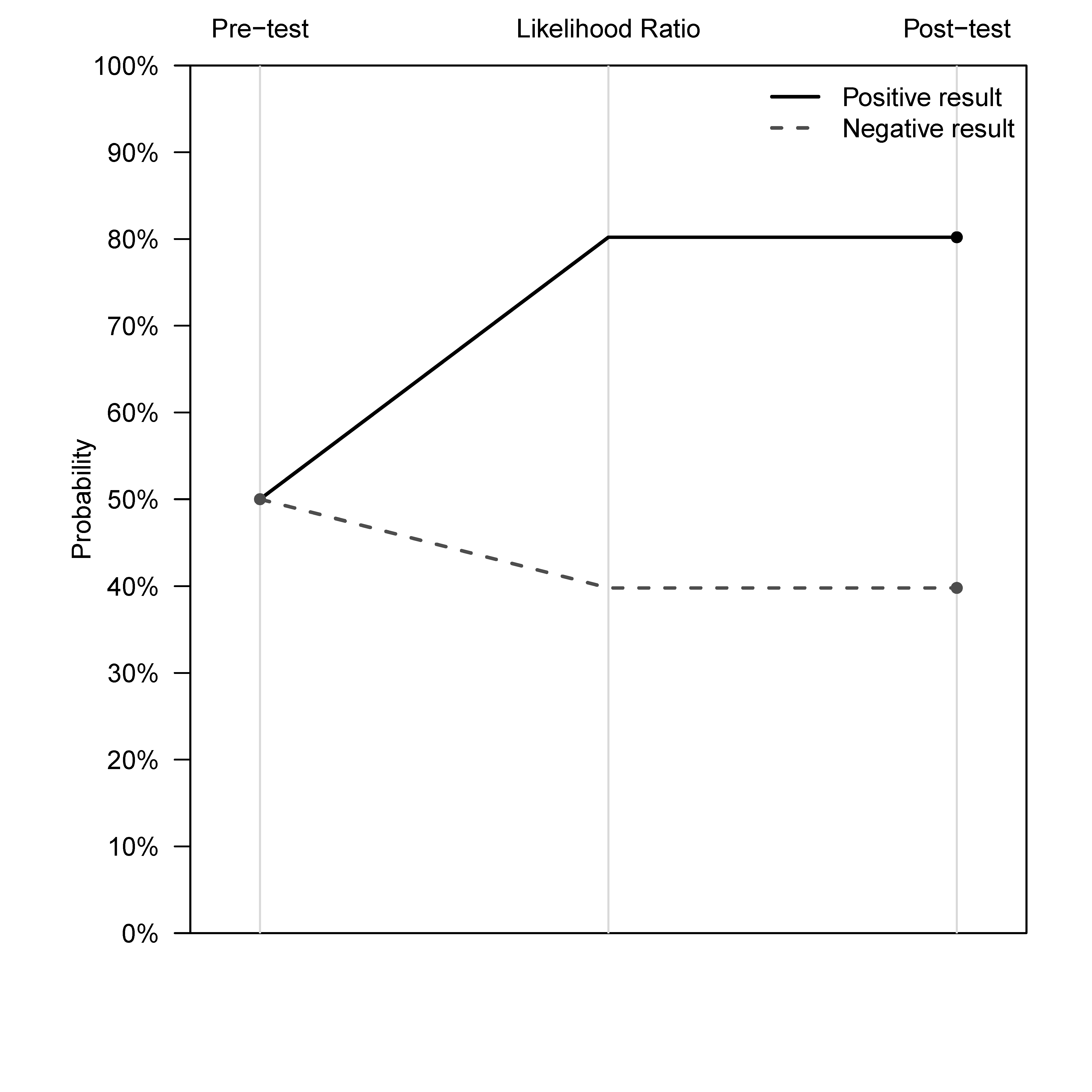

Supplement: otag056_Supplementary_Data [file otag056_supplementary_data.zip › S9_Fagan_nomogram_CD.tif]

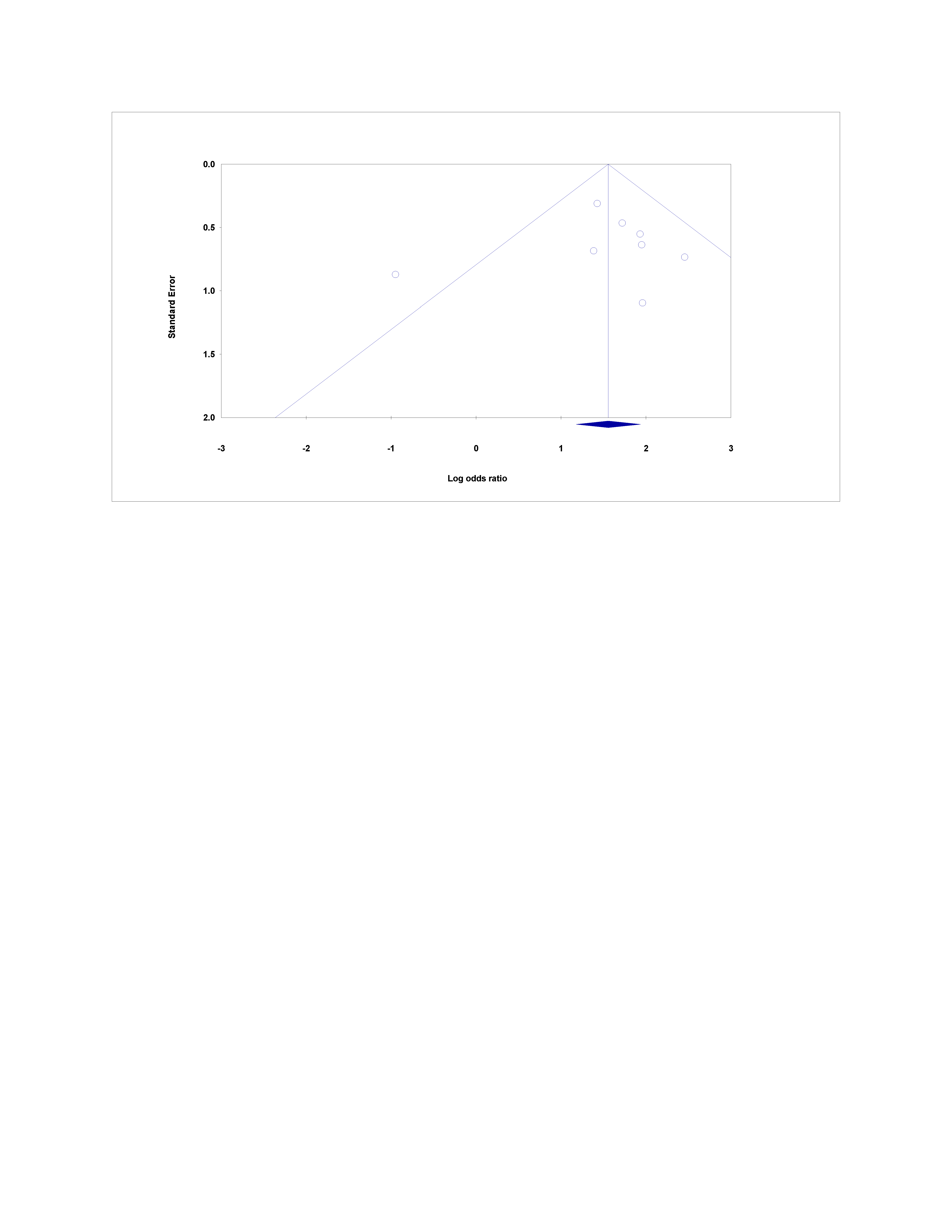

Supplement: otag056_Supplementary_Data [file otag056_supplementary_data.zip › S10. Funnel plot for UC vs controls (8 studies).tif]

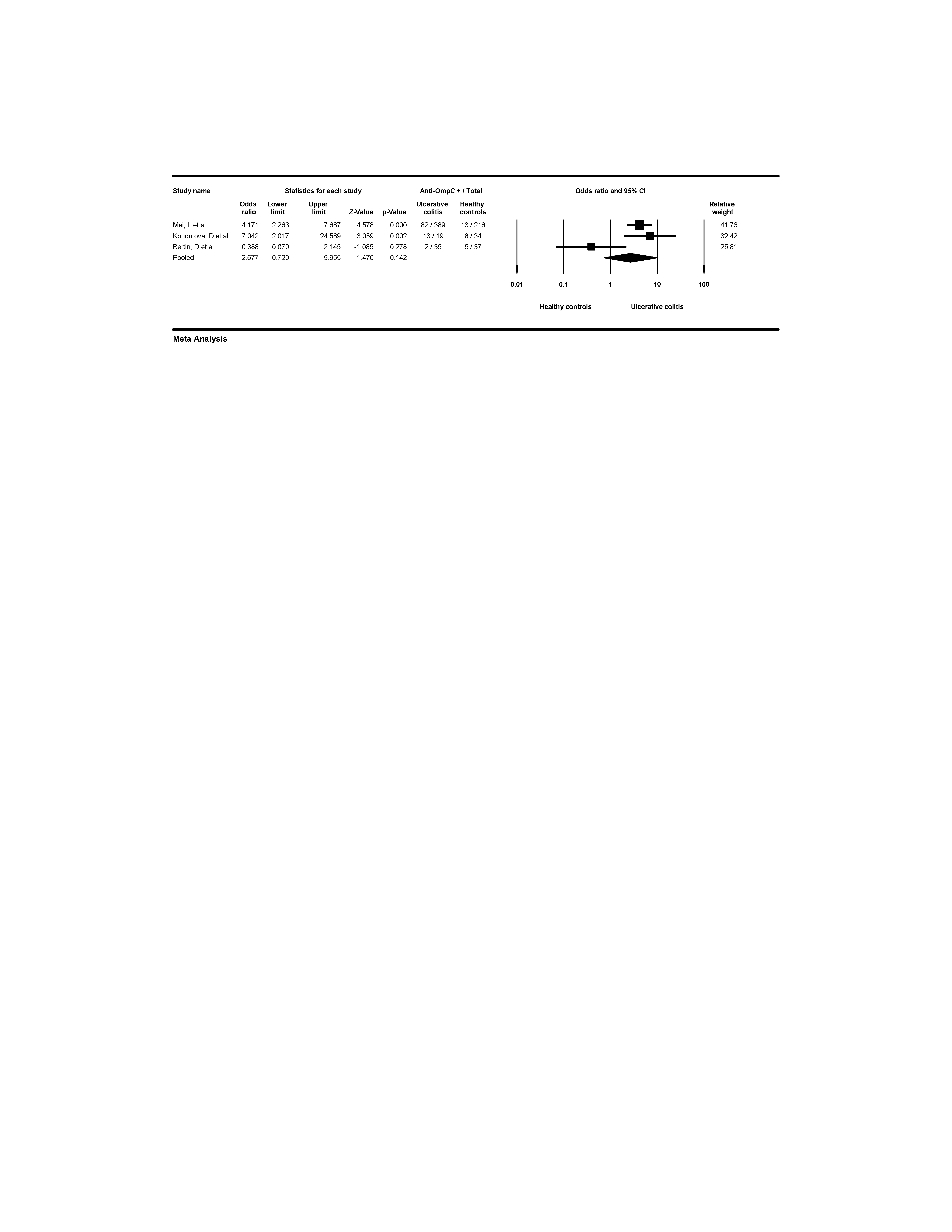

Supplement: otag056_Supplementary_Data [file otag056_supplementary_data.zip › S11. Forest plot OR for UC (high cut off 23-25).tif]

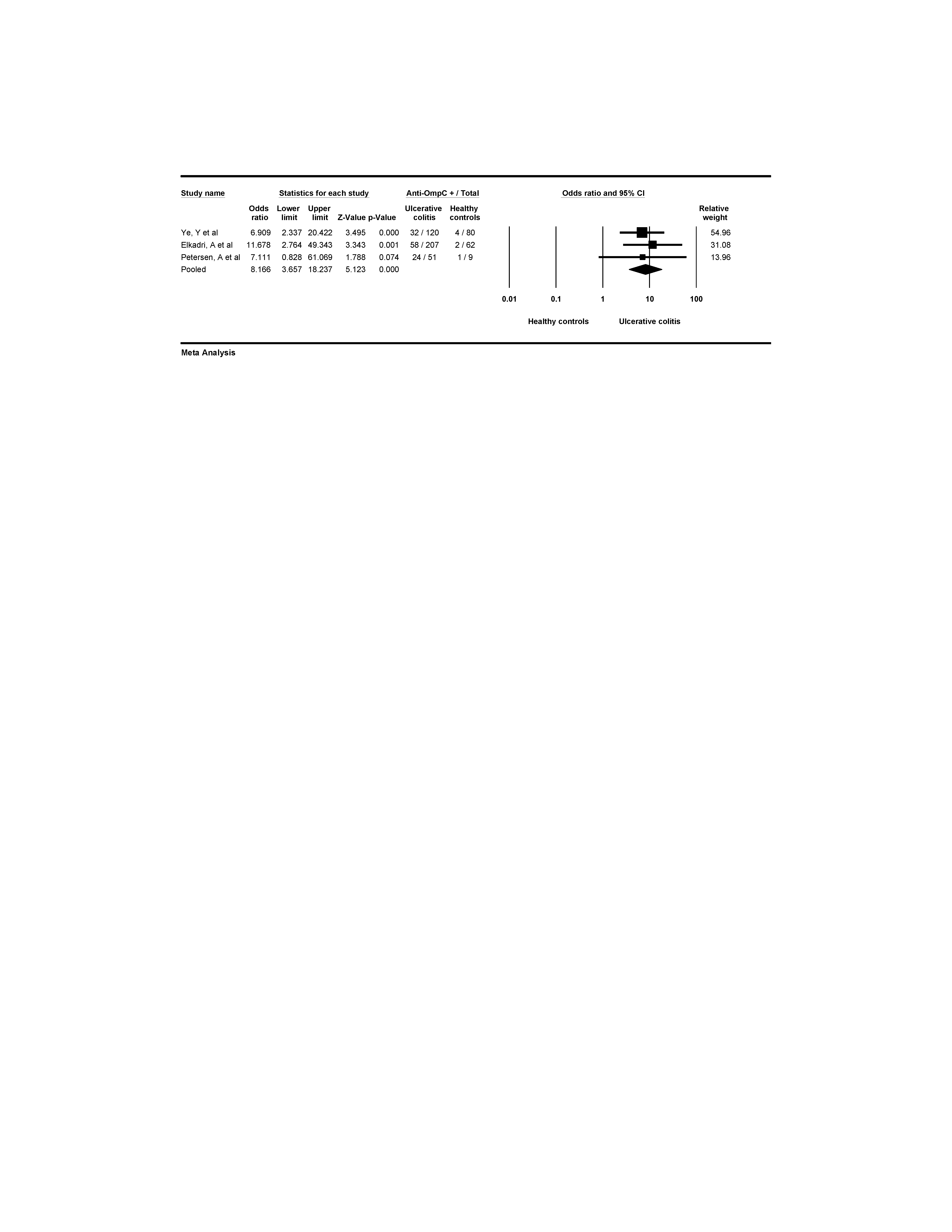

Supplement: otag056_Supplementary_Data [file otag056_supplementary_data.zip › S12. Forest plot OR for UC (low cut off 16-16.5).tif]

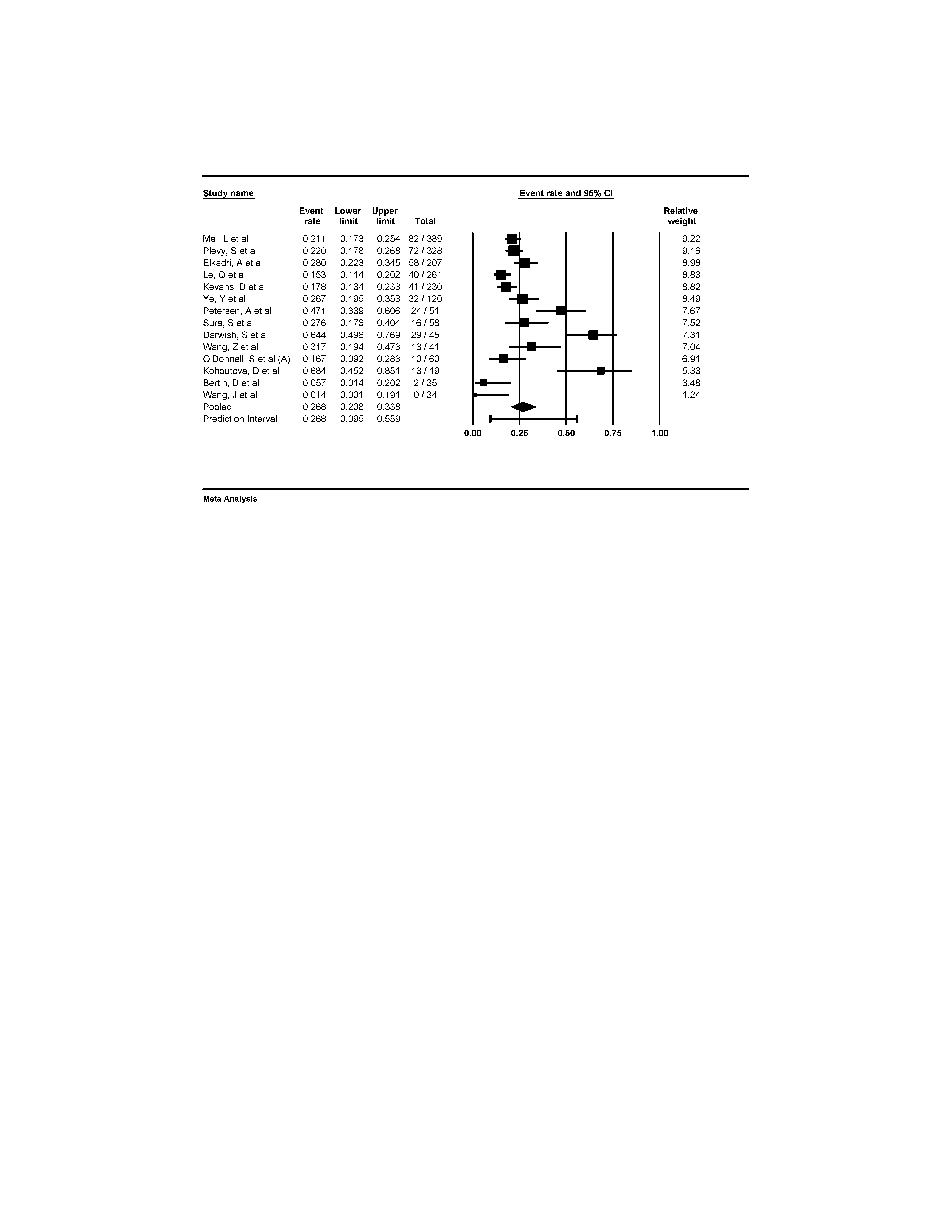

Supplement: otag056_Supplementary_Data [file otag056_supplementary_data.zip › S13. UC prevalence forest plot (All studies).tif]

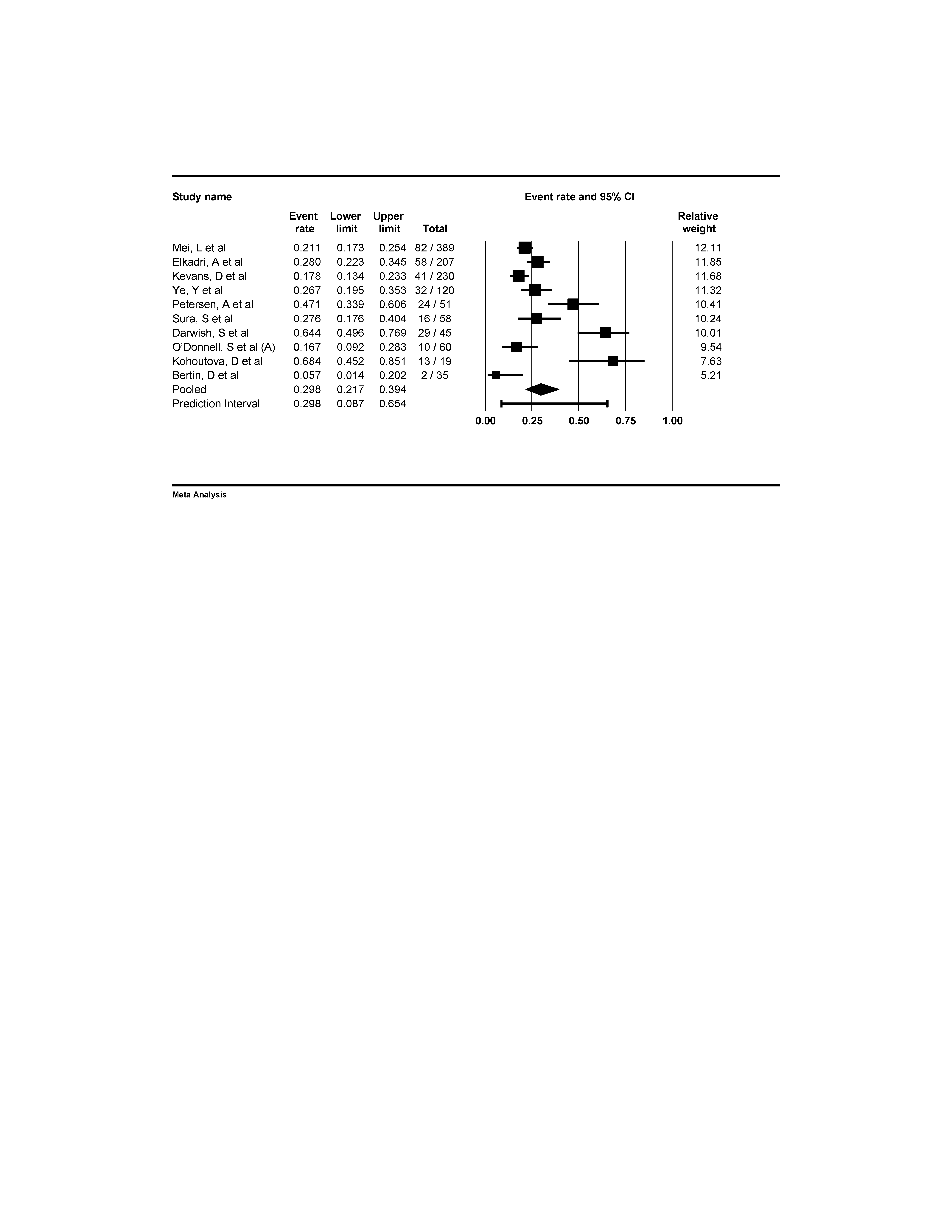

Supplement: otag056_Supplementary_Data [file otag056_supplementary_data.zip › S14. UC prevalence forest plot (numeric 10 studies).tif]

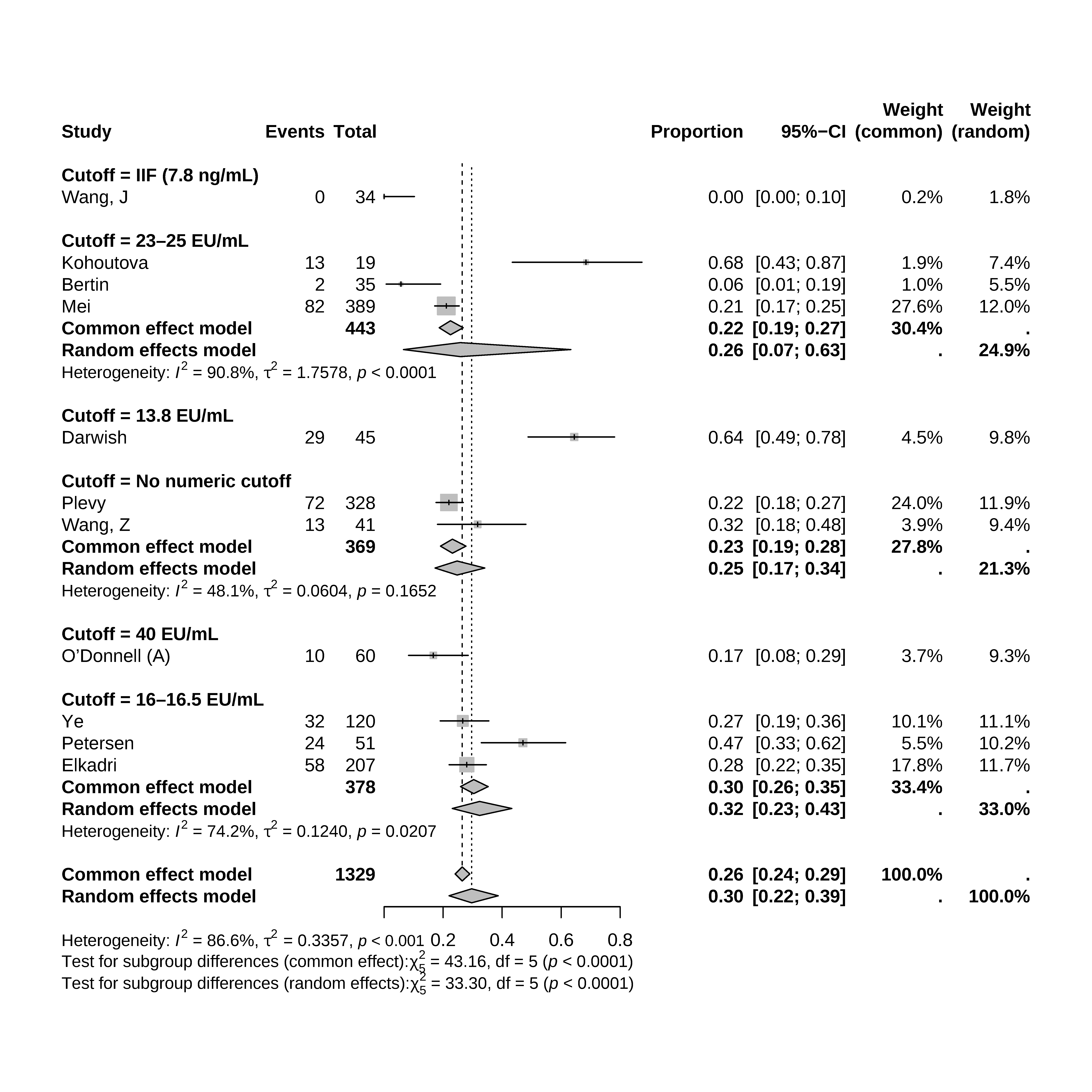

Supplement: otag056_Supplementary_Data [file otag056_supplementary_data.zip › S15. Pooled UC sensitivity (subgroup of 11 studies).tif]

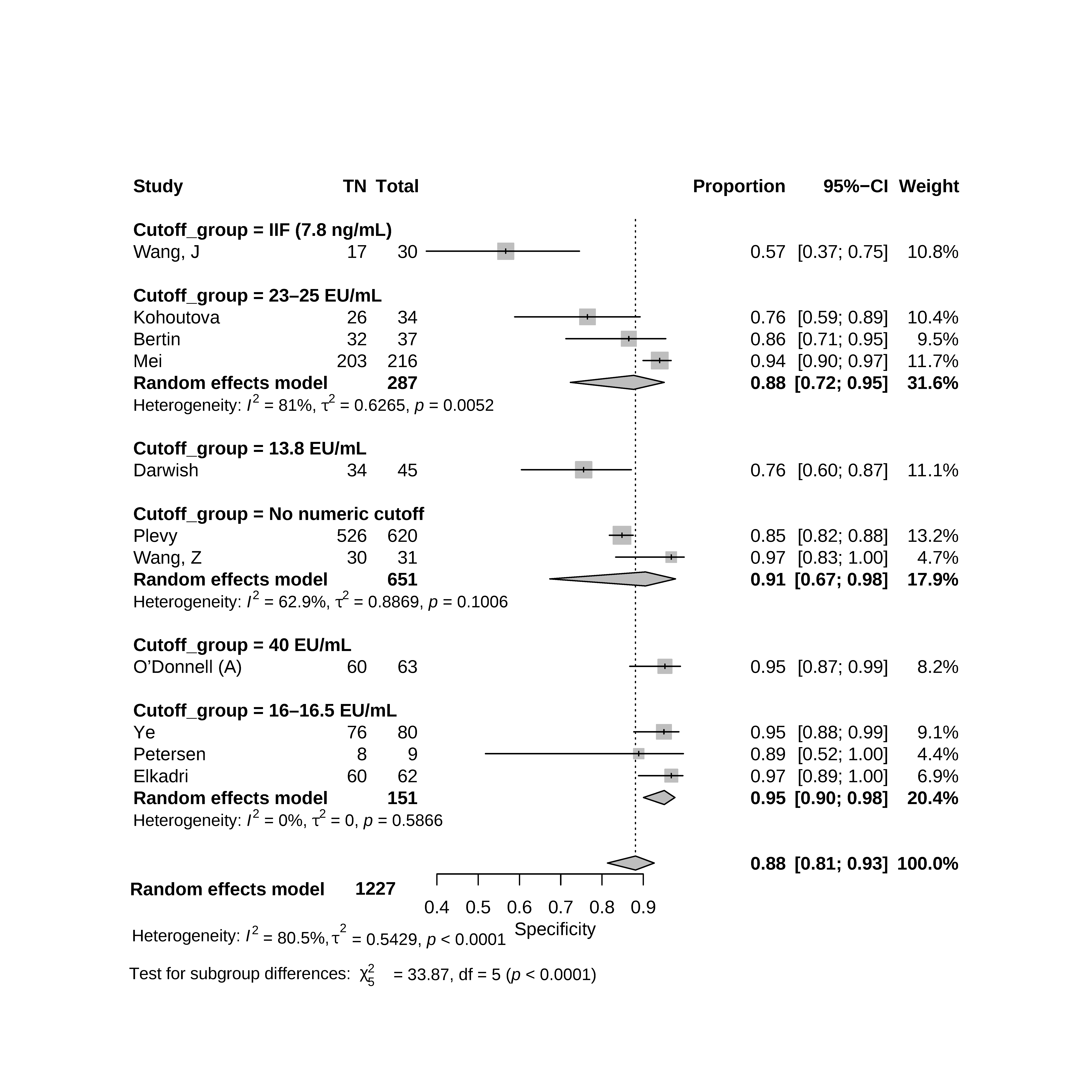

Supplement: otag056_Supplementary_Data [file otag056_supplementary_data.zip › S16. Pooled UC specificity (11 studies, subgroups).tif]

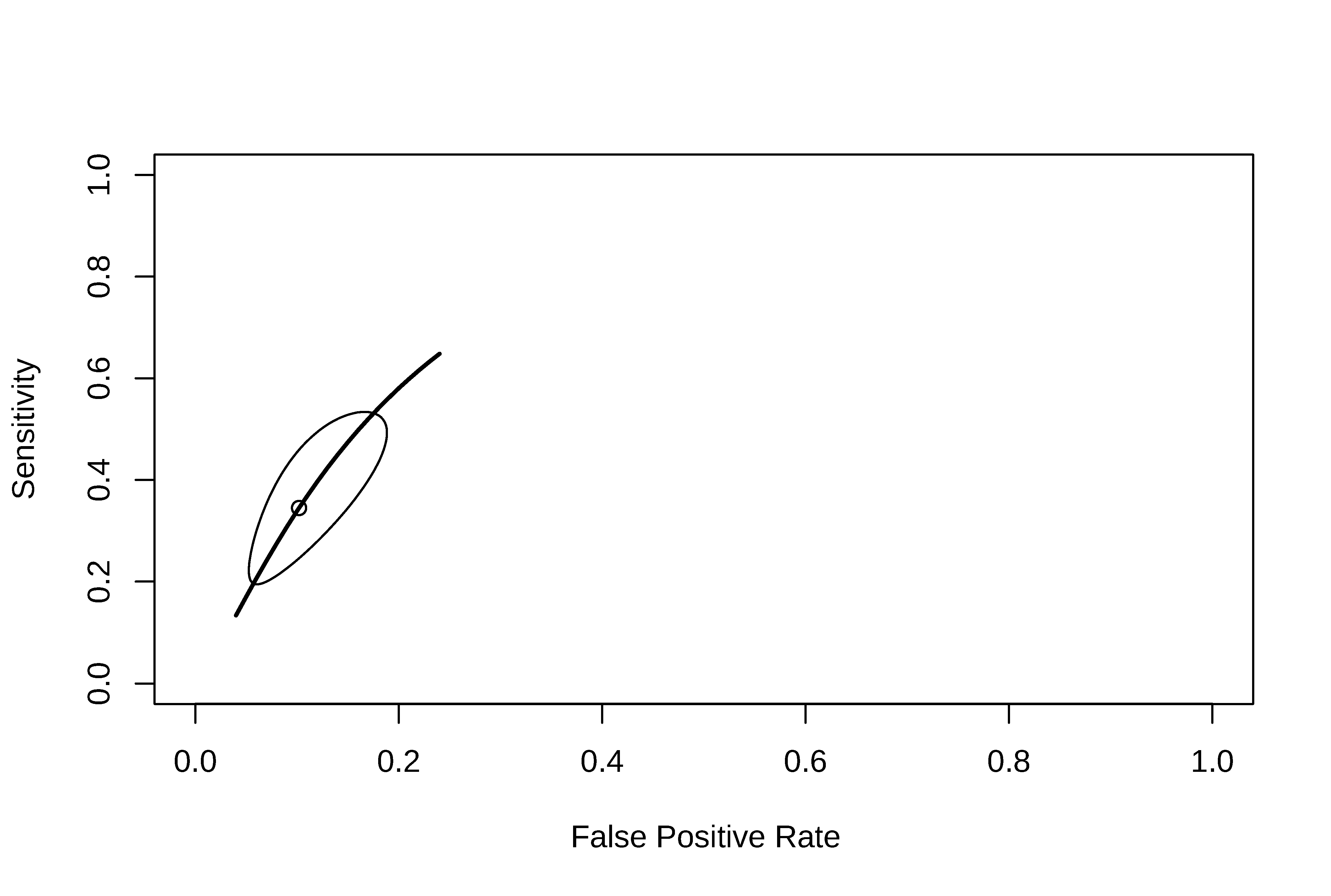

Supplement: otag056_Supplementary_Data [file otag056_supplementary_data.zip › S17. SROC for UC.tif]

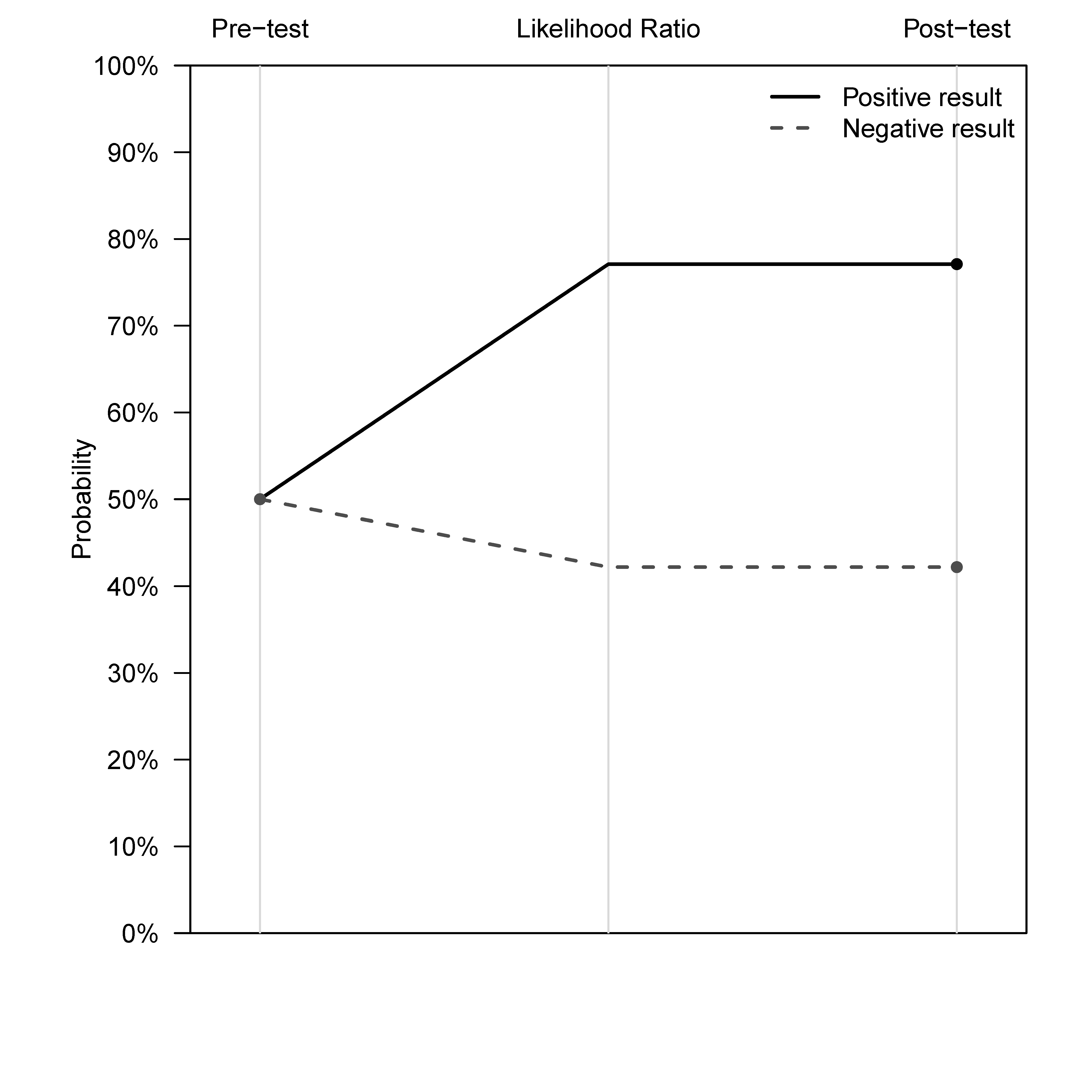

Supplement: otag056_Supplementary_Data [file otag056_supplementary_data.zip › S18_Fagan_nomogram_UC.tif]

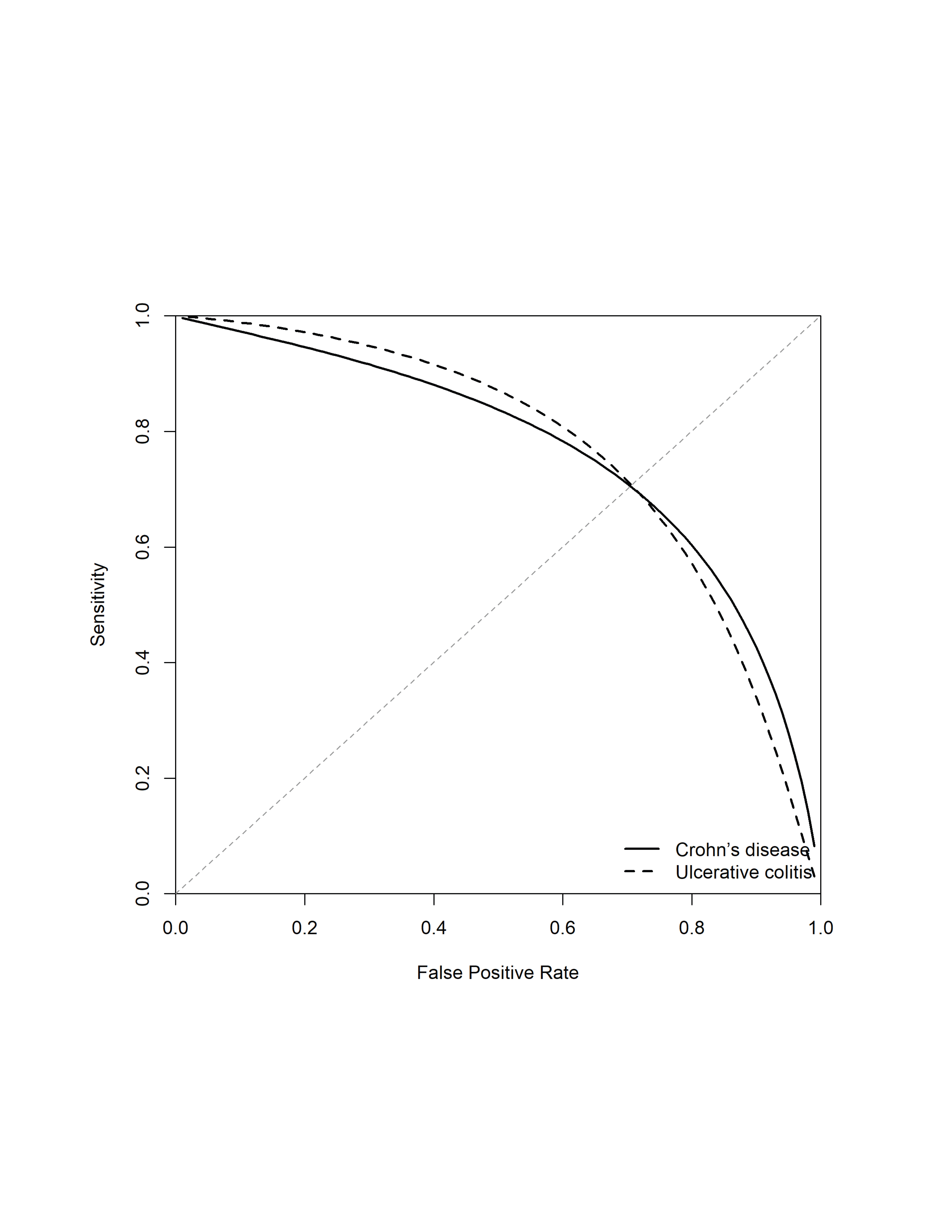

Supplement: otag056_Supplementary_Data [file otag056_supplementary_data.zip › S19. SROC_OmpC_CD and UC combined.tif]

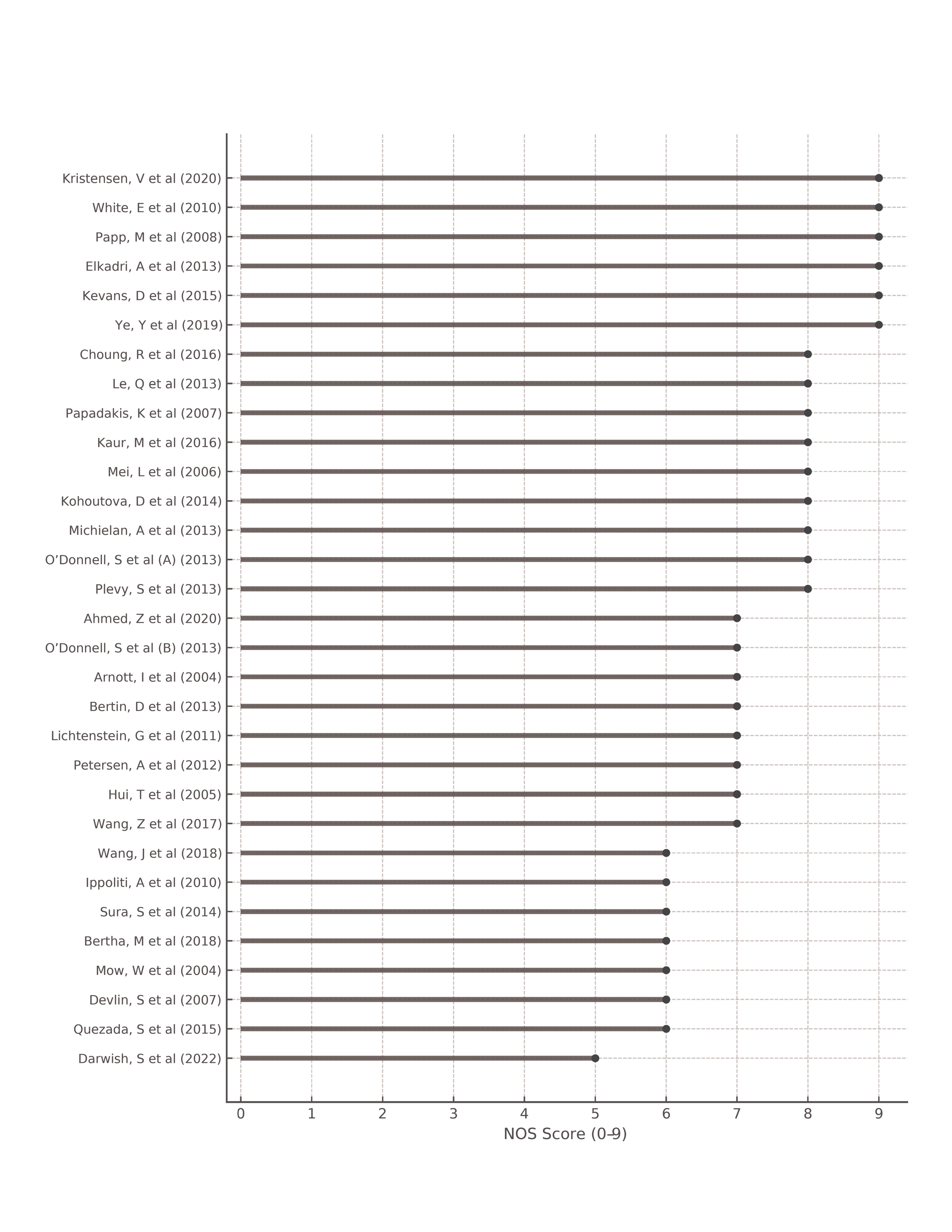

Supplement: otag056_Supplementary_Data [file otag056_supplementary_data.zip › S20. NOS_Quality_Assessment_Figure.tif]

**QUADAS-2 Traffic Light Plot for Diagnostic Accuracy Studies**

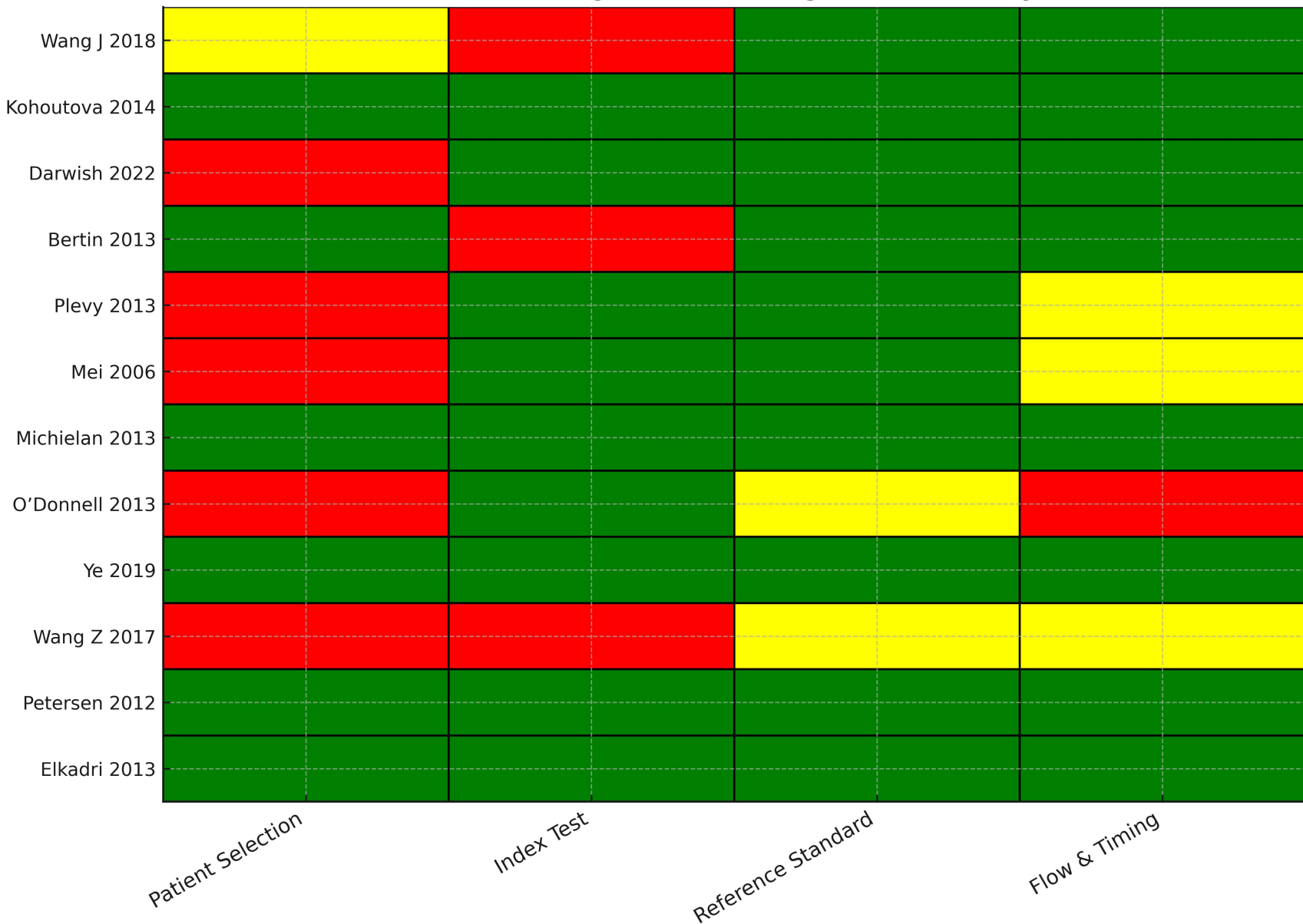

Supplement: otag056_Supplementary_Data [file otag056_supplementary_data.zip › S21. QUADAS2_TrafficLight.pdf]
